# The importance of marshes providing soil stabilization to resist fast‐flow erosion in case of a dike breach

**DOI:** 10.1002/eap.2622

**Published:** 2022-05-17

**Authors:** Beatriz Marin‐Diaz, Laura L. Govers, Daphne van der Wal, Han Olff, Tjeerd J. Bouma

**Affiliations:** ^1^ Department of Estuarine and Delta systems NIOZ Royal Netherlands Institute for Sea Research Yerseke The Netherlands; ^2^ Conservation Ecology Group, Groningen Institute for Evolutionary Life Sciences University of Groningen Groningen The Netherlands; ^3^ Department of Coastal Systems NIOZ Royal Netherlands Institute for Sea Research Den Burg The Netherlands; ^4^ Faculty of Geo‐Information Science and Earth Observation (ITC) University of Twente Enschede The Netherlands; ^5^ Department of Physical Geography, Faculty of Geosciences Utrecht University Utrecht The Netherlands

**Keywords:** ecosystem conservation, ecosystem services, ecosystem‐based coastal defense, flood protection, flow erosion, overtopping, sediment erosion, soil surface erosion, top erosion, wave run up

## Abstract

Salt marshes provide valuable ecosystem services including coastal protection by reducing wave loading on dikes and seawalls. If the topsoil is erosion resistant to fast‐flowing water, it may also reduce breach depth if a dike fails. In this experiment, we quantified the topsoil erosion resistance from marshes and bare tidal flats with different soil types to understand the extent to which they can help reduce breach depth. Intact soil samples were collected from 11 locations in the Netherlands at different tidal elevations and then exposed for 3 h to 2.3 m/s currents. To the samples that remained stable after flow exposure, an artificial crack was made to test their stability following soil disturbance. All samples from the tidal flats were completely eroded, regardless of sediment type. In contrast, all samples from well‐established marsh plateaus were stable as long as no disturbances were made, including those with sandy subsoils. After creating artificial cracks, samples with a thin cohesive top layer on top of sandy subsoil collapsed, while marshes with silty subsoils remained stable. Pioneer marshes on sandy substrate without a cohesive top layer were the only vegetated soils that completely eroded. The lower erosion of marshes with either sandy or silty soils compared to bare tidal flats was best explained by the presence of a top layer with belowground biomass, high organic content, high water content, and low bulk density. When analyzing the erodibility of marshes only, fine root density was the best predictor of erosion resistance. This study demonstrates the importance of preserving, restoring, or creating salt marshes, to obtain a topsoil that is erosion resistant under fast‐flowing water, which helps reduce breach dimensions if a dike fails. The probability of topsoil erosion in established marshes with sandy subsoil is higher than in silty marshes. A silty layer of cohesive sediment on top of the sand provides extra erosion resistance as long as it does not break. Pioneer marshes that have not developed a cohesive top layer are erosion sensitive, especially in sandy soils. For future marsh creations, using fine‐grained sediments or a mixture of sand with silt or clay is recommended.

## INTRODUCTION

Many coastal communities are facing flood risks due to accelerating sea level rise, land subsidence, and intensifying storms, which will probably further increase with climate change (IPCC, 2014; Syvitski et al., 2009). As a result, increasing investment in coastal defense structures is needed worldwide (Temmerman et al., 2013). Combining “green” infrastructure, such as salt marshes or mangroves, with conventional “gray” infrastructures like sea‐walls and dikes, can improve the coastal protection in addition to be a more sustainable solution by preserving natural ecosystems and its related ecosystem services (Morris et al., 2018; Schoonees et al., 2019; Shepard et al., 2011; Temmerman et al., 2013). Furthermore, nature‐based flood defenses may be capable of recovering from storm disturbances (Feagin et al., 2015; Gijsman et al., 2021) and be resilient against sea‐level rise (Fagherazzi et al., 2020; Kirwan et al., 2016; Morris et al., 2020). Ecosystems higher in the intertidal zone like marshes, mangroves, or dunes will have more direct effects on coastal protection (Bouma et al., 2014). For example, salt marshes can effectively reduce waves even under storm surge conditions (Möller et al., 2014; Willemsen et al., 2020) and lower the wave run up on the dikes compared to dikes with bare tidal flat in front (Vuik et al., 2016; Zhu et al., 2020). As a result, it can be shown that the presence of marshes reduce the likelihood of a dike breach during historic storm floods (Zhu et al., 2020). Moreover historic analyses revealed that the presence of a marsh in front of a dike reduced the breach depth by providing an elevated stable soil layer, thereby saving many lives during flooding (Zhu et al., 2020). As this latter effect is becoming increasingly important when having dikes protecting people living in low‐lying areas faced with sea level rise (Zhu et al., 2020), there is urgent need to gain in‐depth understanding of the erosion resistance of foreshores fronting dikes against fast flow running over the soil surface.

**FIGURE 1:**
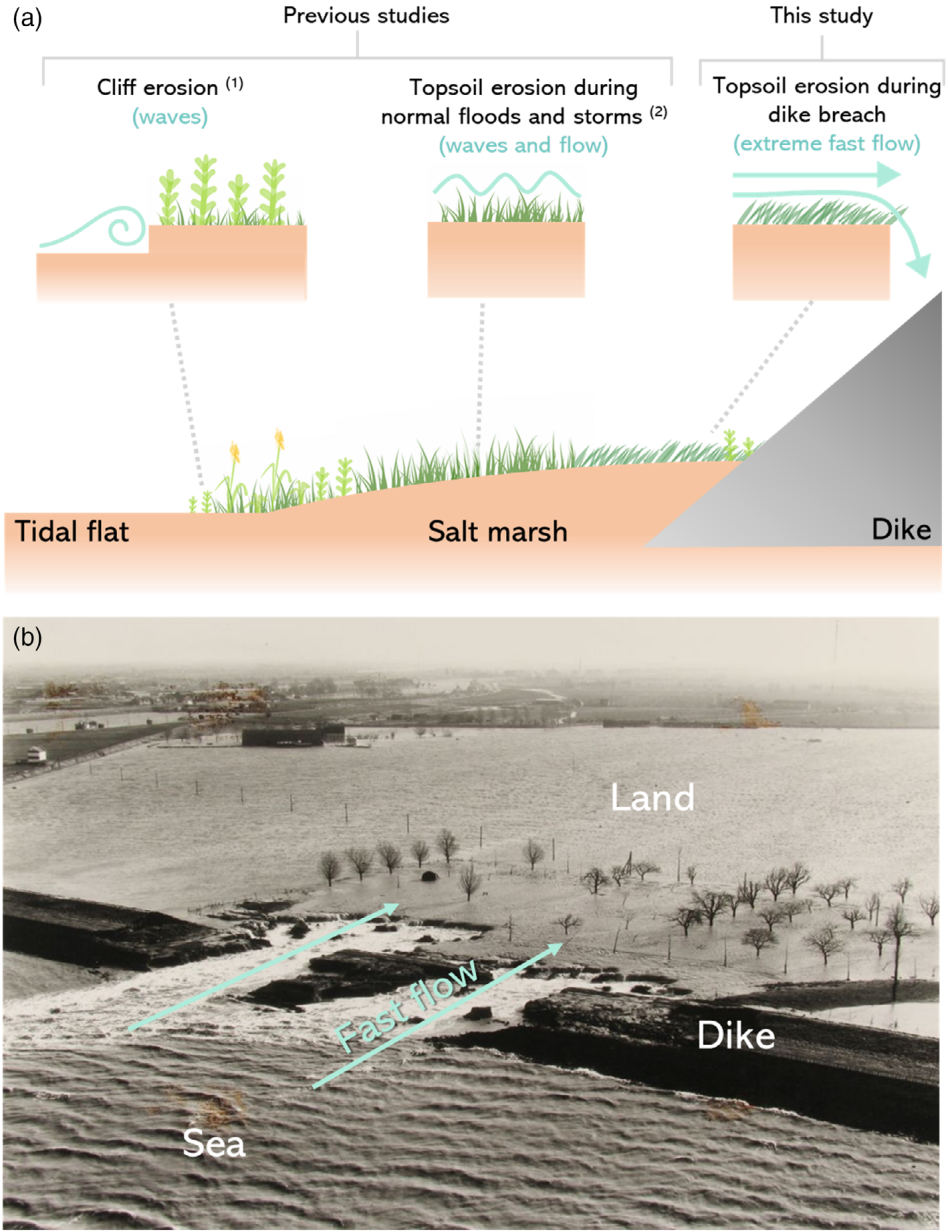
(a) Diagram depicting the types of marsh erosion processes previously studied under controlled conditions: (1) cliff erosion c.f. Feagin et al. (2009), Ford et al. (2016), Lo et al. (2017), Wang et al. (2017), and De Battisti et al. (2019); and (2) topsoil erosion during normal floods and storms c.f. Coops et al. (1996), Bouma, Friedrichs, Klaassen, et al. (2009), Spencer et al. (2016), and Paul and Kerpen (2021). In this study, we focus on a different type of marsh topsoil erosion that can occur during a dike breach: fast‐flow topsoil erosion. (b) Example of fast flow following a dike breach after the winter storm of 1953 in Nieuw‐Neuzenpolder, Zeeuws‐Vlaanderen, Westerschelde Estuary. Photo credit: ZB, Beeldbank Zeeland.

Salt marsh soil stability has mainly been tested regarding lateral or cliff erosion (Figure 1a), as related to marsh retreat (Brooks et al., 2020 and references therein). Fine‐grained soils, higher organic content and/or high belowground biomass have been correlated with less lateral erosion (Feagin et al., 2009; Ford et al., 2016; Wang et al., 2017). More specifically, higher root density has been linked to lower lateral erosion, which becomes increasingly important for sandier soils (De Battisti et al., 2019; Lo et al., 2017). Higher soil salinity has been related to higher belowground biomass (Alldred et al., 2017) and less marsh retreat during hurricanes (Howes et al., 2010). Additionally, large grazers such as livestock can modify the soil properties and belowground biomass, thereby increasing the resistance to lateral erosion (Davidson et al., 2017; Marin‐Diaz et al., 2021; Pagés et al., 2018). The effect of salt marshes on topsoil (surface) erosion resistance (Figure 1a) has, to the best of our knowledge, only rarely been studied under controlled conditions. Specifically, there have been few experiments focusing on the effect of belowground biomass after the removal of aboveground vegetation. Measurements under strong wave conditions after clipping the aboveground vegetation showed that marsh soil with belowground vegetation is highly erosion resistant (Spencer et al., 2016). Coops et al. (1996) also found reduced surface erosion under wave exposure in reed species growing on sandy soils compared to bare soils. Furthermore, the effect of the belowground biomass under wave conditions occurs even with the vegetation in winter state (Paul & Kerpen, 2021). However, in pioneer marsh vegetation, which can be sparse and grow in patches, tidal current can induce surface erosion on sandy substrates due to scouring around stiff stems (Bouma, Friedrichs, Klaassen, et al., 2009; Bouma, Friedrichs, Van Wesenbeeck, et al., 2009).

To better understand how marshes can reduce the breach depth during a dike failure (Figure 1b), which is a different process from the previous erosion experiments done with foreshore ecosystems (Figure 1a), we need to gain more insight into which factors control the resistance of foreshores against topsoil erosion under fast flow conditions. In terrestrial ecosystems, top soil erosion by runoff was reduced with increasing root density compared to bare soils, especially with high fine root density (below 1 or 0.5 mm diameter, depending on the author; Baets et al., 2006, 2007; Burylo et al., 2012; Li et al., 1991). For vegetation that grows on dikes, it was shown that topsoil erosion was also reduced with increased root density together with clay sediments and with higher plant species diversity (Scheres & Schüttrumpf, 2019, 2020 and references therein). However, to our knowledge, which factors control the resistance of foreshores soils against topsoil erosion under fast water flow conditions has not yet been studied.

To further our understanding of the extent to which foreshores, and marshes in particular, can help reducing breach depth, we investigated the topsoil erosion resistance of salt marshes and tidal flats under conditions of fast water flow (2.3 m/s; Figure 1a). More specifically, we focused on the effect of soil and belowground vegetation properties reducing the top erosion. We excluded aboveground vegetation as they might break off, and to get insight into the erosion resistance under the most erosion‐sensitive setting. For this, soil samples of tidal flats and salt marshes representing a wide range of vegetation and sediment types were obtained from elevational transects at 11 intertidal areas around the Netherlands. Erosion was measured following 3 h of exposure to fast water flow in a flow flume. Subsequently, artificial cracks were applied to samples that remained stable to test their stability following soil damage like could happen if debris hit the soil or tension cracks develop during a dike breach.

## MATERIALS AND METHODS

### Study sites

**FIGURE 2:**
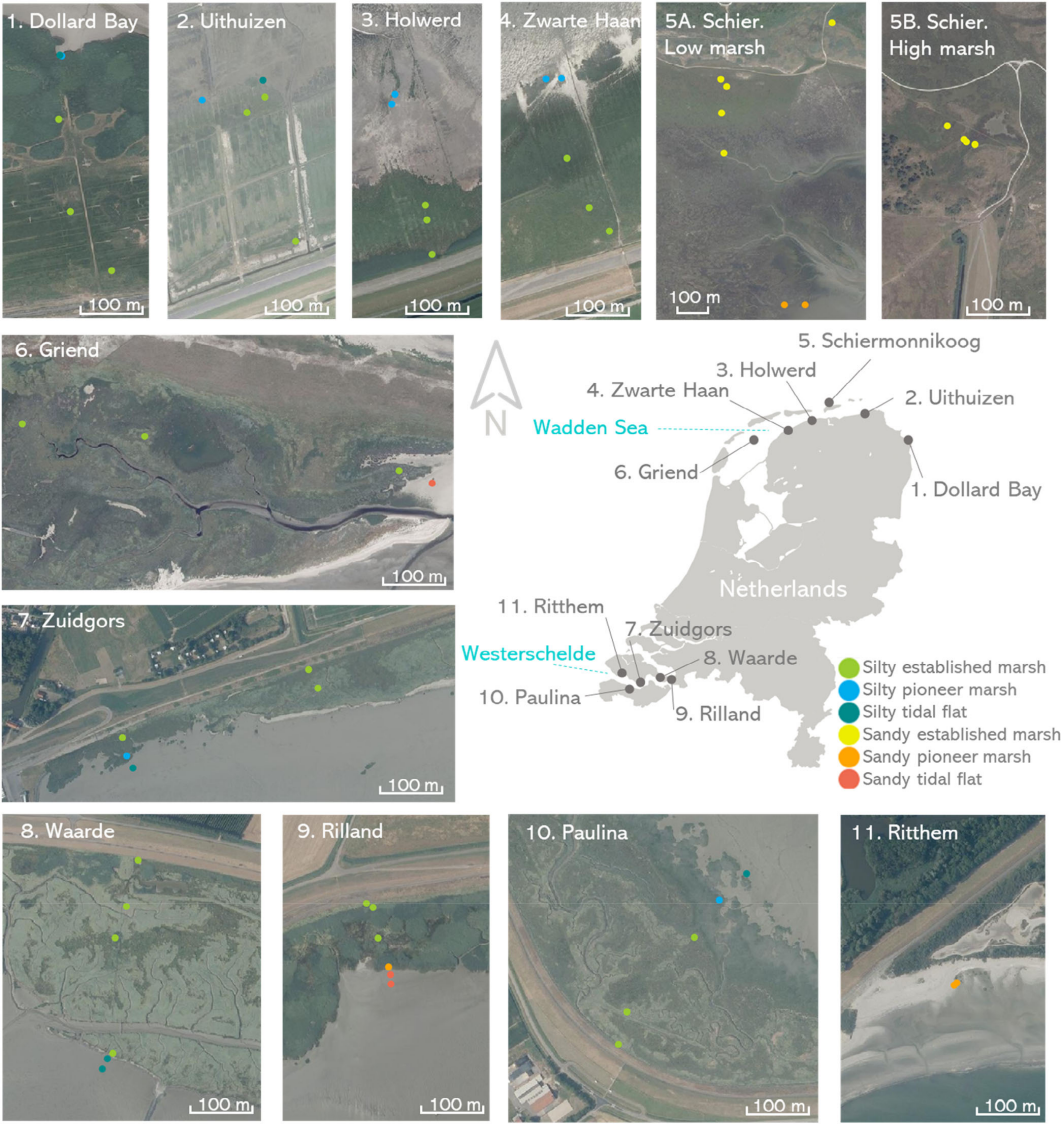
Sampling locations in the Wadden Sea and the Westerschelde estuary. Color points indicate the habitat type, with silty soils in cool colors and sandy soils in warm colors. Photographs were obtained from PDOK (Public Geodata Portal in The Netherlands) and all are oriented with the north pointing up.

Soil samples were collected in salt marshes and tidal flats from 11 locations in the Netherlands to include a wide range of biological and physical conditions (Figure 2). Six locations were located in the Wadden Sea, which is a meso‐tidal zone with a tidal range between 2 and 3 m (RWS, 2013) in the north of the Netherlands (Figure 2). The Wadden Sea sampling areas (Appendix S1: Table S1) included four locations along the Dutch mainland coast (Dollard Bay, Uithuizen, Holwerd, and Zwarte Haan), one barrier island (Schiermonnikoog), and one fetch‐limited barrier island (Griend). The remaining five locations were located along the Westerschelde, the estuary of the Scheldt river with a meso to macro‐tidal range between 4 and 5 m (RWS, 2013) in the southwest of the Netherlands (Figure 2). This Westerschelde sampling area (Appendix S1: Table S1) consisted of both relatively exposed (Waarde, Rilland, and Zuidgors) and relatively sheltered (Paulina Polder and Ritthem) marshes (Callaghan et al., 2010; Van der Wal et al., 2008).

### Experimental set up

The samples were collected along elevation transects to obtain a range of different soil and vegetation types. The transects started in front of the dikes and went through the different vegetation types, ending in the pioneer marsh and the tidal flat (Figure 2, Appendix S1: Table S1). The samples were classified into six habitat types (1) silty established marshes, understanding “established” as the marsh plateau, (2) silty pioneer marshes, found on the edge of the silty mature marshes, (3) silty tidal flats, (4) sandy established marshes, with a sand with peat cohesive top layer and sandy subsoil, (5) sandy pioneer marshes, without cohesive top layer, and (6) sandy tidal flats (Figure 2). Samples were also classified by grazing status of large herbivores like cows and sheep (grazed or ungrazed) and type of surface (cohesive, with thick detritus layer, with cracks due to a summer drought, or with soft mud; Appendix S1: Table S1).

### Top erosion

Rectangular soil cores of 40 cm long × 20 cm high × 13 cm wide were collected in all the study sites during October and November of 2018. The cores were extracted with a custom‐made steel box‐core with sharp edges that was inserted in the soil and carefully dug out, placing a board below the bottom to keep the sample intact (Appendix S1: Figure S1). The soil sample was then carefully placed into a custom‐made wooden box that fits in the flow flume. To prevent cohesive soil samples from getting stuck to the walls of the steel frame, the frame was sprayed inside with a thin layer of oil. The oil did not interfere with the erosion experiment because the parts exposed to the erosion were never in contact with the oil. The wooden boxes had nails in the bottom to prevent the complete sample from dislodging with the water flow. From each rectangular soil core, 8 cm from the total 40 cm length was cut off from one of the sides to analyze the vegetation belowground, leaving a soil sample of 32 × 20 × 13 cm for the erosion test (Appendix S1: Figure S2). The samples were transported to the Royal Netherlands Institute for Sea Research (NIOZ) in Yerseke and stored in tanks with seawater to keep the sediment wet until the erosion test. Muddy samples were covered with a plastic film in the field to prevent drying out during the transport. The elevation from each sampling site was measured with a dGPS (Leica GS12).

**FIGURE 3:**
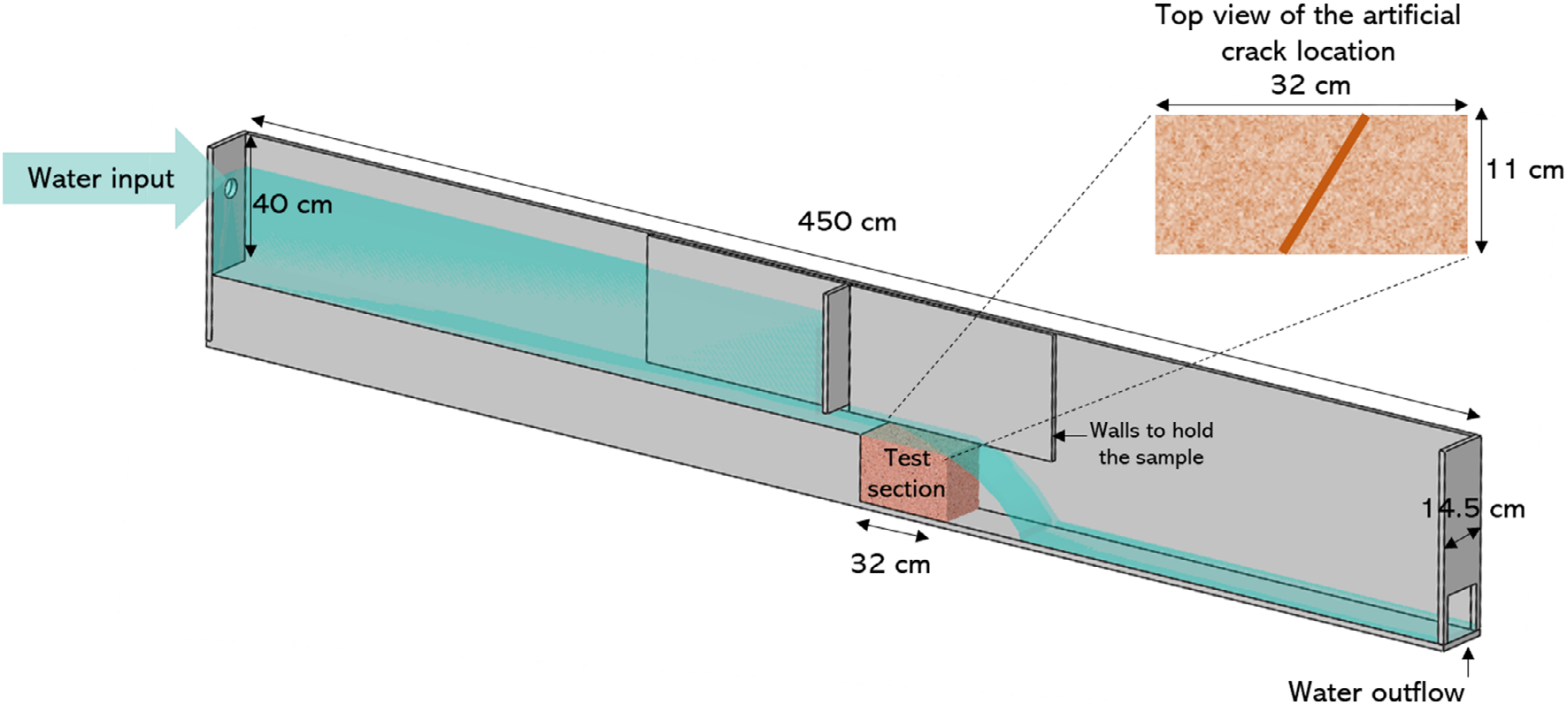
Schematic cross‐section of the fast flow flume developed and built at the Royal Netherlands Institute for Sea Research (NIOZ). In the top right, a diagram of the location in a sample of the artificial cracks made for the second part of the experiment. Water flow reached 2.3 m/s.

Top erosion was determined in a fast flow flume developed in the NIOZ (Figure 3). The flume consisted of a water tank filled at a constant flow rate of 247 L/min. The tank had an opening of 2.4 cm high and 11 cm wide in the bottom of one corner from where the water was flowing at a constant velocity of 2.3 m/s with an estimated bottom shear stress of ~27 N/m^2^ (Appendix S1: Equation S1). Due to the formation of a supercritical sheet flow, bottom shear stress calculations may not be strictly valid but can serve as an estimation. The flow velocity was based on the scenario of a dike breach (Albers, 2014; Kamrath et al., 2006). The samples were placed on the other side of the opening, exposing the top layer of the samples to continuous water flow (Appendix S1: Figure S2). The outflowing water was disposed of. Before each erosion test, the aboveground vegetation was clipped to the soil surface level to remove any erosion‐protective effects of the canopy. Two wooden walls were installed on each side of the test section to avoid water infiltration through the sides of the sample, leaving an exposed area of 32 cm long × 11 cm wide. Top erosion was determined after 10 min, and 1, 2, and 3 h by measuring the average change in elevation with a pin‐profiler adapted from the sedimentation erosion bar method (Nolte et al., 2013) and similarly used in Scheres & Schüttrumpf (2019). Two lines of measurements were done at 3‐cm intervals along the axis parallel to the flow and 3.7 cm spacing on the cross‐stream axis. The vertical resolution was 1 mm. Samples were classified into stable (mean erosion up to ~2 cm depth) and non‐stable (completely eroded). The erosion after 10 min was subtracted from the total mean erosion after 3 h in stable marsh samples to avoid a bias in the results because it was mainly loose debris.

After the top erosion tests, artificial cracks were made to 17 samples that had remained stable to mimic damage in the soil like could occur during a dike breach (e.g., by debris hitting the ground or development of tension cracks). Samples with sandy bottoms and different cohesive layer thickness were included. Two types of cracks were made in the center of the soil samples (Figure 3). The first crack type was 4 cm deep × 2 cm wide and samples were exposed to the water flow for 1 h. If the sample did not collapse, a crack of 8 cm deep × 2 cm wide was made and exposed for another hour. To test if the soil would remain stable after a longer time exposure, six of the samples were exposed to the fast flow for 16 h (Appendix S1: Figure S3). The cracks were always oriented with a 45° angle (Figure 3).

### Belowground vegetation properties

Belowground vegetation properties were determined from an 8 × 20 × 13 cm subsample of the same soil samples collected for the erosion test. The belowground biomass was cleaned and separated into roots and rhizomes. Representative subsamples of rhizomes, coarse roots (>0.5 mm diameter), and fine roots (<0.5 mm diameter), including fine dead root material that could not be distinguished and removed (cf. De Battisti et al., 2019), were separated from each sample to calculate the respective proportions. This separation was done to disentangle the effect of each root compartment on topsoil erosion. The samples and subsamples were dried at 60°C until constant weight to obtain the biomass. Densities of each compartment were calculated as the respective biomass divided by the volume of the soil sample (g/cm^3^; e.g., Baets et al., 2006; De Battisti et al., 2019; Marin‐Diaz et al., 2021).

### Soil properties

Additional small cores of 2.2 cm diameter and 20 cm depth were collected next to each rectangular core to determine the soil properties. The core was split from 0 to 5 cm and 5 to 20 cm depth. In the case of sandy bottoms, the soil was split from 0 cm to the start of the sand layer, and from there to 20 cm. The sediment samples were freeze dried for 4 days. From these samples, we calculated the bulk density as the dry weight in a known volume (g/cm^3^) and the percentage of soil water content as the difference between wet and dry mass (Wang et al., 2017). To determine the soil organic content, first the coarse roots, rhizomes, and large detritus were removed from the sediment to take into account only the particulate organic matter. The sediment was then burned for 6 h at 450°C (Craft et al., 1991). Sediment grain size was determined with a Malvern Mastersizer 2000. Clay‐silt fraction (<63 μm), hereafter called silt content, has been previously related to clay content and soil cohesion in our marine region (Van Ledden et al., 2004) and for reproducibility it was used in this study instead of the clay fraction alone (e.g., Ford et al., 2016; Lo et al., 2017; Marin‐Diaz et al., 2021). For more information on the other soil fractions obtained see Appendix S1: Table S2. Additional deep soil profiles up to 1.5 m depth were extracted to visually explore the presence of deeper sandy layers. Finally, a simple and fast field‐applicable method adapted from Howison et al. (2015) and hereafter referred to as dynamic soil deformation test, was measured to study if it could be used as a proxy for soil erodibility. This method consisted of releasing a 5 kg metal weight of 10 cm diameter 10 times from 1.5 m height through a guiding PVC pipe. The distance between the soil surface and the bottom of the compacted soil (cm) was used as the dynamic soil deformation value.

### Data analysis

Data was first analyzed including all the samples (the ones that completely eroded and the ones that barely eroded). Soil properties from the top layer (0–5 cm depth) were used for the analysis. To visually analyze the nonlinear relationships among environmental variables and their effect on erosion, nonmetric multidimensional scaling (NMDS) analysis based on Bray‐Curtis similarities (a nonparametric multidimensional analysis) and Spearman correlation matrices were used. Stress values reported with the NMDS indicate the accuracy of the representation (stress <0.05 indicates perfect representation, <0.2 should be interpreted with caution and >0.3 indicates arbitrary ordination; Clarke, 1993). Because the probability of complete erosion including all the samples was binary (completely eroded or not eroded, Appendix S1: Figure S4), correlations between the environmental variables and complete erosion probability were modeled with logistic generalized linear models (GLM) with binomial distribution. To find the best combination of environmental variables explaining the complete probability of erosion, an initial logistic GLM was built including only the total belowground biomass, rhizome density, soil organic content, percent silt, mean grain size, and dynamic soil deformation. The initial variables were selected based on the NMDS and correlation matrices to avoid collinearity. For instance, total root density, total belowground biomass, and fine root density were strongly correlated (Appendix S1: Figure S5). Soil water content, organic content, and bulk density were also strongly correlated (Appendix S1: Figure S5). The final model selection was based on stepwise regression and the lowest Akaike's information criterion (AIC). The significance of the models was tested using a Type II Wald χ^2^ test. Additionally, simple logistic regressions were fitted to each individual variable to visualize their correlation with the complete erosion probability. A limitation from these simple logistic regressions was that the significant trend in silt and mean grain size seemed to be due to the higher number of silty vegetated samples.

To study the variability in erosion among stable samples, a second analysis was done using only the samples that remained stable, now using the erosion‐depth (cm) as continuous variable. Because of the nonlinear relationships among variables, the same procedure of NMDS ordination based on Bray‐Curtis similarities and Spearman correlation matrices were used to visually analyze the relationships among environmental variables and their effect on erosion. Gamma with link log GLM were fitted to each environmental variable and erosion depth to assess the possible correlations within stable samples.

The erosion with artificial cracks was modeled fitting a logistic GLM with binomial distribution, using the binary erosion (collapse or not collapse) as the response variable and the ratio of cohesive layer depth to crack depth as the independent variable. All statistical analyses were performed using R 3.5.0 (R Development Core Team, 2018). The *vegan* package was utilized for the NMDS ordinations (Oksanen et al., 2018).

## RESULTS

### Top erosion in different habitat types

**FIGURE 4:**
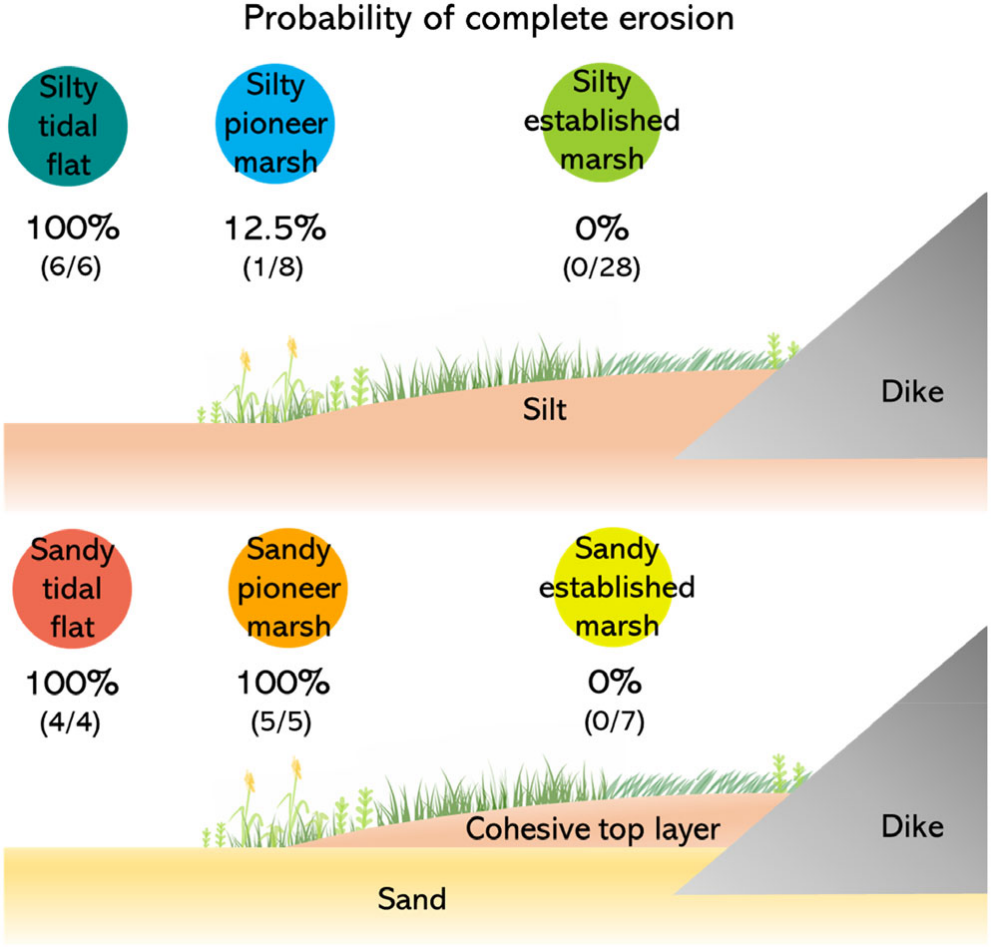
Graphic representation of the habitat types and the probability of complete erosion in samples from each of these habitats. Numbers in brackets indicate the number of samples completely eroded by the total number of samples for each habitat type.

After 3 h of flow exposure, samples were found to be either completely eroded or not eroded (up to 2 cm), hereafter called stable samples (Appendix S1: Figures S4, S6 and S7). The probability of being completely eroded could be differentiated between habitat types (Figure 4). Silty established marshes, silty pioneer marshes, and sandy established marshes were stable, with the exception of one silty pioneer sample that completely eroded (Figure 4; Appendix S1: Figures S4 and S6). Tidal flats, either silty or sandy, and sandy pioneer marsh samples were all completely eroded (Figure 4; Appendix S1: Figures S4 and S6).

### Relationships between environmental factors and erosion comparing all the samples

**FIGURE 5:**
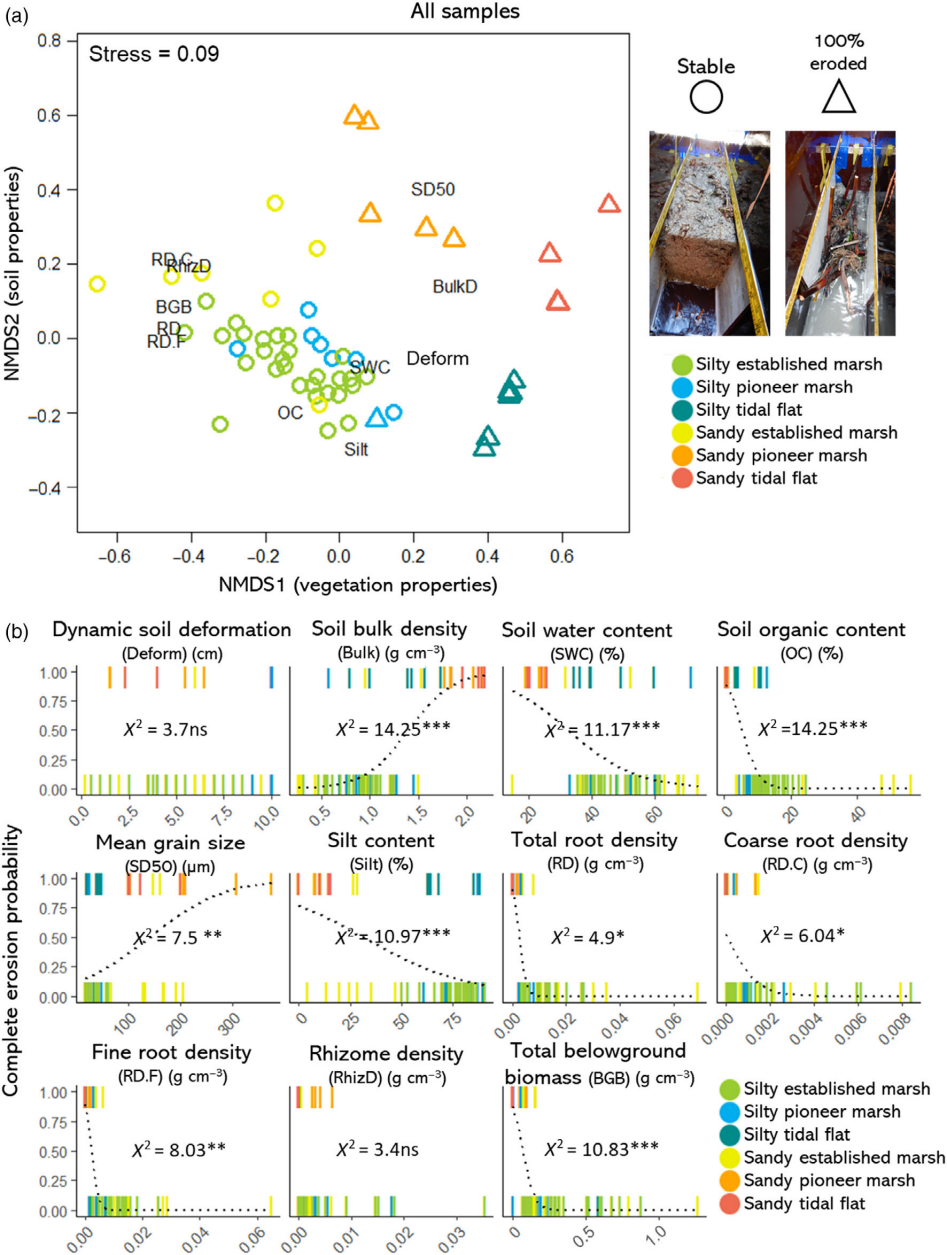
(a) Nonmetric multidimensional scaling ordination depicting the environmental variables, habitat types, and erosion, including samples that completely eroded and stable samples. Erosion is expressed as binary in the shapes of the points. Stress value = 0.09. (b) Simple logistic regressions between the environmental variables and binary erosion (stable = 0 vs. completely eroded = 1) including all the samples. Points are represented as lines for better visualization due to points overlapping. Significance codes refer to *p* < 0.001 (***); *p* < 0.001 (**); *p* < 0.01 (*); *p* > 0.05 (ns).

The NMDS analysis resulted in clear differences between habitat types and erosion types based on the biotic and abiotic factors measured (stress value = 0.09; Figure 5a). Erosion was explained mainly by the belowground vegetation variables (i.e., first NMDS axis) in combination with the soil properties (i.e., second NMDS axis; Figure 5a). In summary, bare tidal flats were completely eroded independently of whether they were silty or sandy (Figure 5a, b). Stable silty soils with vegetation (established and pioneer marsh) had roots and higher organic content in some of the samples compared to silty tidal flats, with the exception of one pioneer sample with very sparse *Salicornia* (Figure 5a,b). In this case, soil water content and bulk density did not strongly differ compared to silty tidal flats (Figure 5a,b). Stable sandy soils with vegetation (established marsh) had higher organic content, lower bulk density and higher water content on the top layer compared to sandy pioneer marsh and tidal flats (Figure 5a,b). In contrast, sandy pioneer marshes (completely eroded) had low belowground biomass in combination with very low organic content, high bulk density, and low water content, similarly to the sandy tidal flats (Figure 5a,b).

The most parsimonious model explaining erosion included total belowground biomass and mean grain size, without significant interaction (GLM: χ^2^[2] = 56.7, *p* < 0.001). Although not selected in the model, there was a significant correlation between lower organic content, higher bulk density, and lower water content with higher erosion, but mainly driven by the sandy samples (Figure 5b). Additionally, we assumed that silt content and mean grain size alone appeared significantly correlated to erosion because of the higher number of silty established marsh samples, rather than because the silt or grain size alone decreased erosion (Figure 5b). Dynamic soil deformation and rhizome density were not correlated to erosion (logistic regressions both not significant; Figure 5b).

### Relationships between environmental factors and erosion comparing only stable samples

**FIGURE 6:**
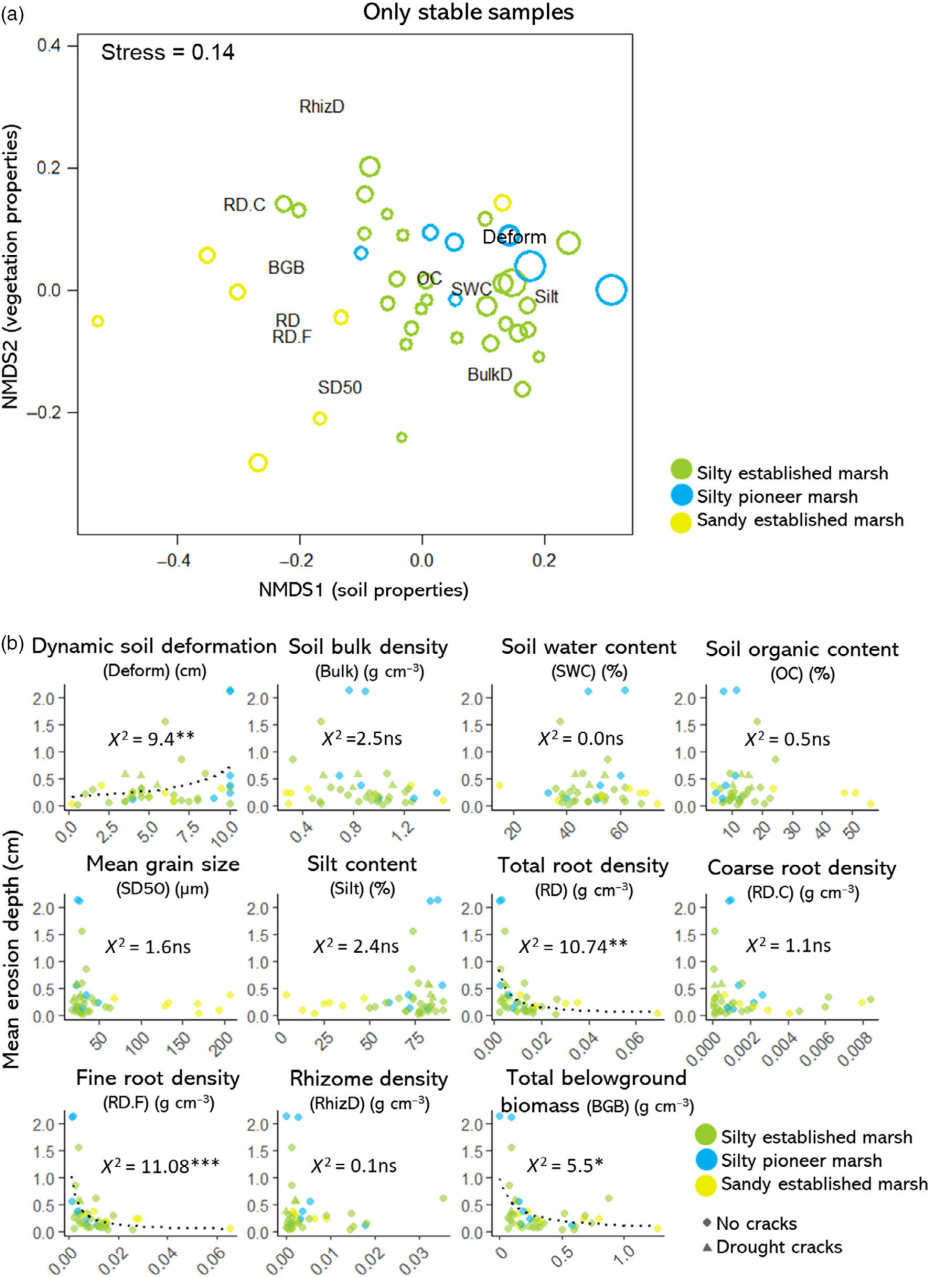
(a) Nonmetric multidimensional scaling ordination depicting the environmental variables, habitat types, and erosion, using only the stable samples. Stress value = 0.14. Erosion is expressed as the size of the points. (b) Simple gamma regressions between the environmental variables and erosion as a continuous variable, with only stable samples. Significance codes refer to *p* < 0.001 (***); *p* < 0.001 (**); *p* < 0.01 (*); *p* > 0.05 (ns).

Comparing only stable samples, the NMDS analysis did not result in a clear separation of the mean erosion depth (cm). Nevertheless, a relationship between root density, dynamic soil deformation and erosion was observed along the first NMDS axis (Figure 6a). Higher fine root density, followed by higher total root density (strongly correlated to fine root density), higher belowground biomass and lower dynamic soil deformation were significantly correlated to less erosion (Figure 6b). The combination of low root density and higher dynamic soil deformation led to the highest erosion, and was found in soft muddy pioneer samples and swampy areas. Additionally, higher erosion was also found in samples with natural cracks due to the summer drought (not captured by the continuous variables measured; Appendix S1: Figure S8 and Table S1) and samples with a thick detritus layer containing *E. atherica* (captured by the dynamic soil deformation test; Appendix S1: Table S1). Rhizome density, coarse root density, and any of the soil properties besides dynamic soil deformation were not significantly correlated with the erosion (Figure 6b).

### Stability of stable marsh samples when artificial cracks were created

**FIGURE 7:**
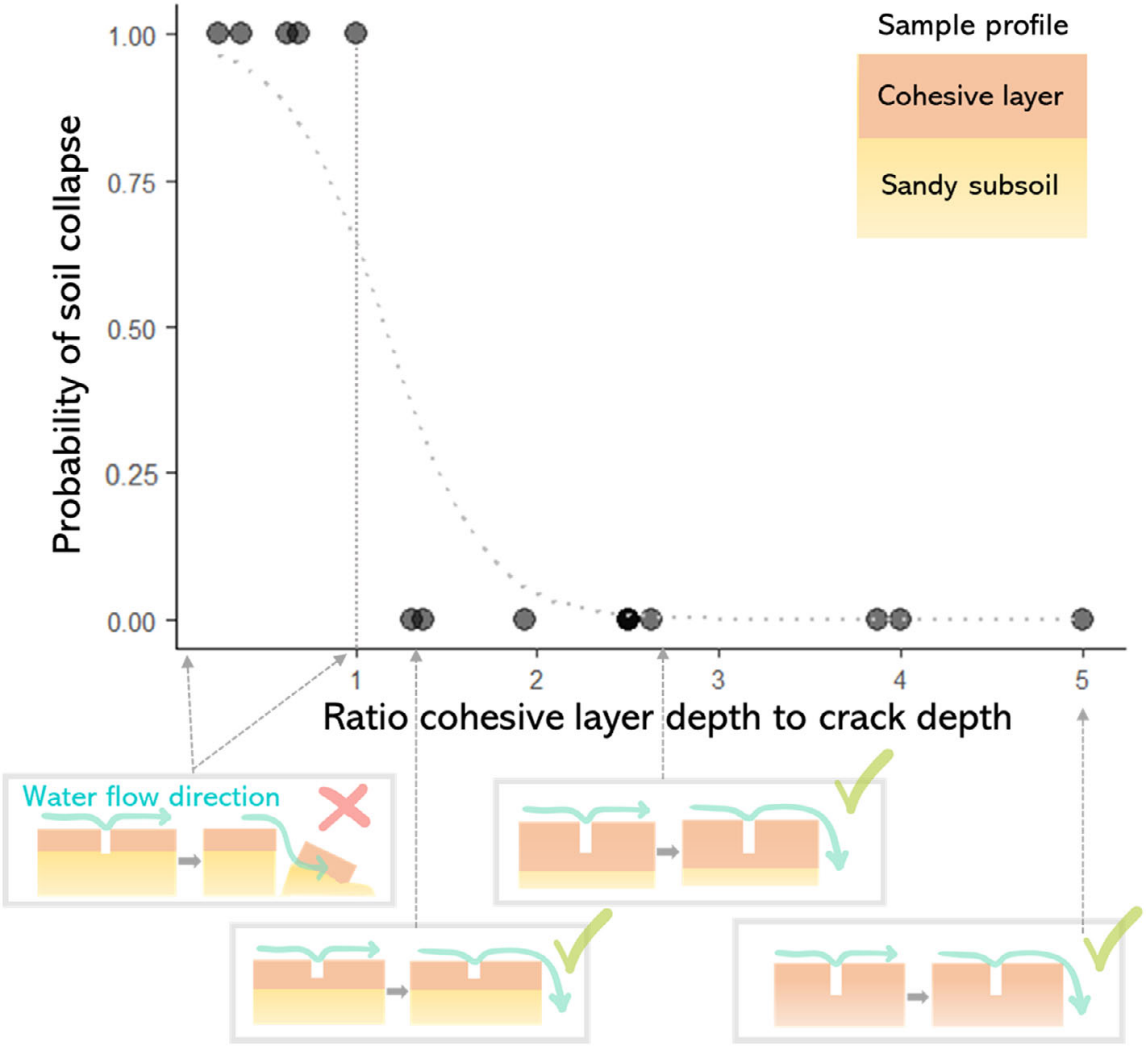
Soil stability in marsh stable samples when creating artificial cracks. Cracks were either 4 or 8 cm deep. Ratios <1 represent cracks that reached the sandy subsoil, therefore representing samples with (1) cohesive layers thinner than 4 cm and cracks of 4 cm or (2) cohesive layers thinner than 8 cm and cracks of 8 cm.

Established marshes with thin cohesive layers (<20 cm, less than the sample depth) and sandy subsoils, were stable when no cracks were present (Figure 3). Nevertheless, when a physical disturbance was artificially created and reached the sandy bottom, they would collapse (Figure 7). With the specific cracks created (4 and 8 cm depth), only the samples with cohesive layers thicker than 8 cm were stable (Figure 7). This process was modeled as the collapse probability explained by the ratio of the cohesive layer depth to the crack depth (GLM: χ^2^[1] = 13.3, *p* < 0.001). When the crack reached the sandy subsoil (ratios ≤1) the samples collapsed, while with ratios above 1, where the crack did not reach the sand, samples were stable (Figure 7).

## DISCUSSION

Understanding the stability of salt marshes is crucial for being able to integrate them into coastal defense schemes (Bouma et al., 2014). Most erosion experiments on marshes have focused on the erodibility of cliffs to understand marsh width (e.g., see De Battisti et al., 2019; Feagin et al., 2009; Ford et al., 2016; Lo et al., 2017; Wang et al., 2017), while only few studies have focused on the erodibility of surface/topsoil under wave forcing (Coops et al., 1996; Paul & Kerpen, 2021; Spencer et al., 2016; Figure 1a). To gain more insight into which marsh types can help reduce breach depth in case of a dike failure, we investigated the factors determining marsh erodibility under fast water flow as can occur during a dike failure (Albers, 2014; Kamrath et al., 2006). We found that established salt marshes even with sandy subsoils were resistant to fast water flow (2.3 m/s), compared to tidal flats, which completely eroded, independently of their sediment properties. Pioneer sandy marshes were the only marsh type that were completely eroded. Silty pioneer marshes were mostly stable, with the exception of sparse *Salicornia* with very low root density (which could be classified as pre‐pioneer). Some of the established marshes had a thick root mat and erodible sandy subsoil, as described by Allen (1989). These samples were stable if the cohesive top layer was intact, but collapsed as soon as an (artificial) crack reached the sandy subsoil.

### Effect of belowground vegetation and soil properties on top soil erosion

Interestingly, although silty and sandy established salt marshes had different soil properties, both were equally stable compared to bare tidal flats. This indicates that, in the bigger picture, established marsh vegetation is what made the foreshores stable in the first place, although the underlying processes varied among habitat types. We did not find any correlation between the soil type and vegetation species, as all the species were found in both sandy and silty marshes, with the exception of *Juncus maritimus* (only sampled in Schiermonnikoog, which was sandy) and *Phragmites australis* (only found in silty locations; Appendix S1: Table S1). Overall, the factors controlling topsoil erosion in bare versus vegetated soils were similar to the findings on lateral erosion, where the roots and organic content reduce erosion and sand content increases erosion (De Battisti et al., 2019; Feagin et al., 2009; Lo et al., 2017). However, in our study, a high percentage of sand increased erosion only in pioneer vegetation, and not in established marshes. In the sandy pioneer marshes, which, although being vegetated, were completely eroded, the cohesive top layer of fine roots, organic matter, and/or silt, found in the stable marshes, was missing. These samples were differentiated by having higher bulk density and lower water content than the stable marsh samples, due to the coarse grain size and very low organic content present (Bartholdy et al., 2010). Lower erosion in established sandy marshes was correlated with higher belowground biomass, lower bulk density, higher organic content, and higher water content in the top layer, and in some cases, silt content was slightly higher than in the bare sandy soils. Hence, in the sandy vegetated samples with <25% silt, similar in bare sand samples, higher organic content together with the belowground biomass may have played the biggest role reducing erosion, similarly to Feagin et al. (2009). In the samples with higher silt content in the top layer compared to the sandy tidal flats, which in our study region is correlated to the clay content and therefore sediment cohesion (Houwing, 1999; Van Ledden et al., 2004), may also have reduced erosion together with the belowground biomass and organic content. In contrast, vegetated silty soils (either pioneer or established marsh) had similar physical properties to silty tidal flats with slightly higher organic content only in some vegetated samples but not in all. Therefore, the primary factor reducing erosion in silty soils may be the belowground biomass and secondarily the organic matter, similar to Lo et al. (2017). The dynamic soil deformation test used in this study was not a good proxy for erosion when including all the habitat types such as tidal flats and sandy marshes without cohesive top layers.

When analyzing the differences of erosion within samples that remained stable (up to 2 cm of total mean erosion), we found the fine root density (<0.5 mm diameter) as the most important factor, independent of the grain size. This is in agreement with terrestrial experiments on top erosion both in silty (Baets et al., 2007; Burylo et al., 2012; Li et al., 1991) and sandy soils (Vannoppen et al., 2017). This means that a high density of fine roots would be more effective at reducing erosion than a few coarse roots. However, we have to take into account that coarse roots in this study (maximum 1.6 mm diameter), are still considered fine roots in other studies, where the threshold is at <1 mm diameter (Baets et al., 2007; Li et al., 1991). In contrast, Battisti et al. (2019) found the coarse roots and rhizomes in *Spartina* as the root compartments explaining most of the lateral erosion. This might be explained by the species studied (*Spartina* and *Atriplex*), which have more coarse biomass than other marsh species, so that the belowground biomass will be mainly explained by rhizomes and/or coarse roots. In this experiment, fine root density explained most of the total root density, which includes fine and coarse roots (*r* = 0.99, *p* < 0.001). Therefore, total root density would also be a good indicator of susceptibility to top erosion, similarly to lateral erosion (Marin‐Diaz et al., 2021). Previous topsoil erosion experiments show similar root densities at which erosion starts to be reduced (~5 kg/m^3^; e.g., Baets et al., 2006, 2007; Scheres & Schüttrumpf, 2019). However, one should be careful with attributing the effect to this specific root density because soil properties and erosion tests were different between studies. Total belowground biomass may not be as good of a predictor for top erosion because it includes the weight of the rhizomes, which were not correlated to erosion. For example, soil with high total belowground biomass due to thicker rhizomes but low root density may have higher erosion than a sample with lower total belowground biomass but higher root density. Nevertheless, total belowground biomass is still a significant indicator in this study and in previous lateral erosion experiments (Ford et al., 2016; Lo et al., 2017; Wang et al., 2017).

Higher dynamic soil deformation was also significantly correlated to higher erosion in stable samples. This was driven by few samples with thick detritus layers, found with *Elytrigia atherica*, and in muddy samples on the silty marsh edge (silty pioneer marsh) or swampy areas, all having higher dynamic soil deformation. Additionally, livestock grazing may contribute to lower erosion by compacting the soil (Elschot et al., 2013; Keshta et al., 2020; Marin‐Diaz et al., 2021; Pagés et al., 2018). However in this study, grazing does not seem essential to further reduce surface erosion because similar low erosion values were obtained both in grazed and ungrazed marshes (Appendix S1: Table S1). Last, future research could investigate the critical flow velocities at which stable marshes start to have high erosion.

### Effect of artificial cracks and deeper cohesive soil layers on soil stability

Although in this experiment all the established marshes were resistant to gradual particle‐by‐particle erosion, during a dike breach deeper soil layers may be also exposed to water flow (Zhu et al., 2020). Our small‐scale test with artificial cracks led to block failure in samples with sandy bottoms. This only occurred for marshes with thin cohesive top layers over sandy subsoils, when the artificial crack penetrated to the sandy subsoil. The cohesive layer depth in marshes can increase with age, productivity, sediment availability and flooding frequency (e.g., Olff et al., 1997; Elschot et al., 2013; Koppenaal et al., 2021). Furthermore, grazing can also affect soil accretion, making this process very context dependent (Elschot et al., 2013; Koppenaal et al., 2021). Cracks in the field may be bigger and deeper, therefore even with thicker cohesive layers the marsh could collapse if the sandy subsoil is eroded. This indicates that in case of a dike breach, marshes with sandy subsoils may not be as stable as silty marshes. Similar processes have been reported in the field, due to wave undercutting of the sandy bottoms (Allen, 1989). Nevertheless, even in case of a sandy bottom, the remaining cohesive top layer could still provide more protection than a bare tidal flat. Similarly, previous studies show that tension cracks on the edge of silty soils can lead to bank failure (Allen, 1989; Francalanci et al., 2013). Therefore, although in our study the silty marshes were stable even with artificial cracks, this effect may vary dependent on the crack size. In addition, soil profiles of up to 1.5 m depth from this study indicate that sandy layers can also be found below silty soils, together with a decrease in the amount of roots with increasing depth (Brooks et al., 2020 and references therein; Appendix S1: Figure S9). Therefore we expect the effect of soil disturbances to vary depending on the size of the crack and the depth and type of the exposed soil. Overall, in case of a dike breach, marshes should provide more stability than tidal flats, and silty marshes more than sandy marshes.

### Potential effect of climate change on top erosion: Natural drying cracks

Cracks in marsh soils can occur naturally during summer due to soil shrinkage after low rain periods or lower summer spring tides (Brooks et al., 2020 and references therein). Four silty samples in this experiment had natural cracks due to soil shrinkage after an unusually dry summer in The Netherlands. The cracks were ~4 cm deep and led to higher erosion in the form of blocks compared to soils with similar properties but without cracks. The increased erosion only occurred on the surface of the soil, and not in the layers deeper than the crack depth. Similar processes on a larger scale have been described in the context of cliff erosion formation where tension cracks after soil shrinkage appear in silty marsh edges leading to bank failure (Allen, 1989). In the case of a dike breach, small cracks due to soil shrinkage in the upper marsh like those found in our study may not be very important because they were shallow and only the top layer eroded faster. However, if droughts or heat waves followed by storms become more frequent due to climate change (IPCC, 2014; Perkins‐Kirkpatrick & Lewis, 2020; Vousdoukas et al., 2018), marshes may become less resilient and more fragmented (Cahoon et al., 2011; Derksen‐Hooijberg et al., 2019; Silliman et al., 2005), and the enhanced erodibility seen in this experiment could contribute to marsh degradation.

### Management implications for coastal protection

Overall, to protect the dikes and hinterland, we should aim to conserve existing marshes, even if they are sandy, as far as they have a cohesive top layer. This is not only offering protection by providing increased soil stability, but also wave and flow attenuation (Leonardi et al., 2018; Möller et al., 2014; Zhu et al., 2020). The growth of pioneer marshes should be promoted to become more stable and wider. The creation of new marshes would be the next step, although this is only possible in locations with suitable conditions for marsh establishment (van Loon‐Steensma, 2015). The establishment of new marshes or the expansion of already established marshes can be promoted with the use of groynes and sedimentation fields to increase the soil elevation (Bakker, 2014) or by sediment disposal from dredging activities (Baptist et al., 2019). New restoration approaches using biodegradable artificial structures may also facilitate marsh establishment by reducing physical stresses such as hydrodynamics (Temmink et al., 2020). If the aim is to have a stable soil in case of a dike breach, we discourage the use of sand to create marshes, because sandy marshes will probably take longer to become stable as they need to grow developed roots, accumulate decayed organic matter and accrete a silty top layer to become stable. Therefore, we recommend that the input of sediment should have a fine grain size to provide a stable marsh bottom. In sandy marshes, the placement of a thin layer of fine‐grained sediment amendments to increase soil stability could be investigated, however the potential ecological implications should also be considered (e.g., species composition). Additionally, vegetation with dense root networks could be transplanted to enhance soil stability. A combination of species may be the best option to promote high root biomass (Ford et al., 2016).

## CONCLUSIONS

Overall, we conclude that marshes are much more resistant to topsoil erosion compared to bare tidal flats. This translates into better protection by soil stabilization in the case of a dike breach, preventing the breach from becoming wider. Within the different types of marshes, silty mature marshes will be the most stable. It is not recommended to rely on narrow pioneer marshes, especially with sandy soils, due to the less cohesive sediment and their higher probability of erosion. We should also not rely on mature marshes with sandy subsoils because they could easily erode if the water reaches deeper layers. Nevertheless, in this latter case, the cohesive mat of roots on top of the sand could still provide more protection to the dike than a bare tidal flat. That said, to relate these results to less extreme situations, it should be taken into account that (1) the fast flow velocities used in this experiment do not normally occur in natural conditions (Bouma et al., 2005; Callaghan et al., 2010; Le Hir et al., 2000; Van der Wal et al., 2008) and therefore any type of continuous marsh will likely provide protection against top erosion during normal flooding conditions compared to bare tidal flats, and (2) the aboveground vegetation that was removed in our experiment in order to study the most erosion‐sensitive setting, will normally provide extra erosion protection by flow and wave attenuation (Nepf, 2012). Finally, when creating marshes artificially, we encourage the use of fine sediment inputs rather than erodible sand.

## AUTHOR CONTRIBUTIONS

Beatriz Marin‐Diaz, Laura L. Govers, Daphne van der Wal, Tjeerd J. Bouma, and Han Olff conceived the ideas and designed methodology; Beatriz Marin‐Diaz collected and analyzed the data; Han Olff contributed to data analysis; Beatriz Marin‐Diaz led the writing of the manuscript. All authors contributed critically to the drafts and gave final approval for publication.

## CONFLICT OF INTEREST

The authors declare no conflict of interest.

## Supporting information


Appendix S1

## Data Availability

Data and the scripts used to perform the analysis (Marin‐Diaz et al., 2022) are available from the 4TU. Research Data Repository: https://doi.org/10.4121/15104343.
