# Supplementary material for: The importance of marshes providing soil stabilization to resist fast‐flow erosion in case of a dike breach
Source: Ecol Appl. 2022 May 17;32(6):e2622. doi: 10.1002/eap.2622 (PMC9541637; doi:10.1002/eap.2622)
Supplement: Supplementary file 1 — Appendix S1 [file EAP-32-e2622-s001.pdf]

Supporting information. Marin-Diaz, B., L. L. Govers, D. van der Wal, H. Olff, and T. J. Bouma. The importance of marshes providing soil stabilization to resist fast-flow erosion in case of a dike breach. Ecological Applications.

Appendix S1

Eq. S1. Calculation bottom shear stress and flow velocity 2

Table S1. Sites and properties of the sampling locations 3

Table S2. Grain size samples 5

Fig. S1. Images from field sampling 7

Fig. S2. Custom-made wooden boxes with soil sample 8

Fig. S3. Mean erosion depth for a prolonged time period (16h) in samples with artificial cracks 9

Fig. S4. Mean erosion depth (cm) at the end of the experiment after 3 h of flow exposure 10

Fig. S5. Spearman correlation matrix from the environmental variables 11

Fig. S6. Example of the erosion from sandy tidal flat vs sandy marsh with cohesive top-layer 12

Fig. S7. Examples of erosion profiles 13

Fig. S8. Example of the erosion in form of blocks in samples with natural cracks due to a summer drought 14

Fig. S9. Deep soil profiles (1.5m) 15

Eq. S1. Calculation of the bottom shear stress and flow velocity applied to the samples during the erosion tests. It should be taken into account that the uncertainty in the bed roughness in not possible to measure and that we are dealing with a supercritical sheet flow, for which shear stress calculations may not be strictly valid. Therefore with this calculation we aimed to provide an indication of the shear stress.

Shear stress (N m^{-2}) was calculated as

$$\tau_b = \rho g \ (\frac{u^2}{C^2})$$

Where ρ is water density (1025 kg m^{-3}), g is gravitational acceleration (9.81 m s^{-2}), u is flow velocity and C is the Chézy coefficient (m $^{0.5}$ s^{-1}).

Flow velocity was calculated as

$$u = \frac{d}{h_2}$$

Where d is the discharge, calculated as

$$d = h_0 h_2 \ (\frac{2\,g}{h_2 + h_0})^{0.5}$$

Where h_0 is the water depth of the basin (0.3 m), and h_2 is the minimum water depth behind the gate (i.e., where velocity is highest), calculated as

$$h_2 = \frac{h_1}{contract}$$

h_1 is the opening height (0.024 m) and *contract* is the contraction constant (0.6).

The Chézy roughness coefficient, which can be approximated for hydraulic rough flow as (van Rijn, 2019), was calculated as

$$C = 18\ log_{10}(\frac{12\,h2}{k_s})$$

Where k_s is bed roughness (0.0005 m).

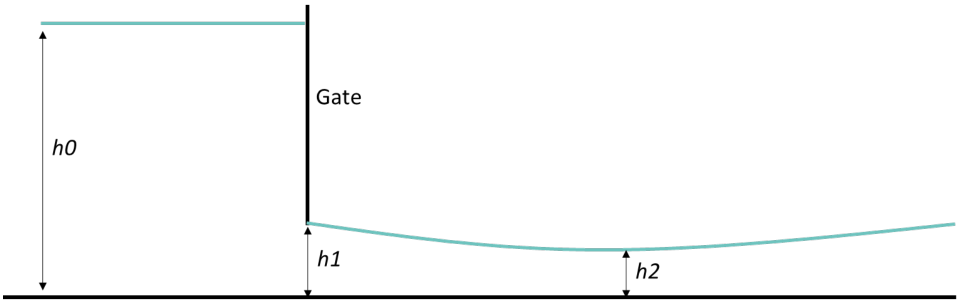

Reference:

van Rijn, L. C. (2019). Erodibility of Mud–Sand Bed Mixtures. *Journal of Hydraulic Engineering*, *146*(1), 04019050. https://doi.org/10.1061/(asce)hy.1943-7900.0001677

Table S1. Sites and properties of the sampling locations. Dollard Bay, Holwerd, Schiermonnikoog, Griend, Uithuizen and Zwarte Haan belong to the Dutch Wadden Sea area. Paulina, Rilland, Ritthem, Waarde and Zuidgors belong to the Westerschelde area in the South of the Netherlands.

	Code sample	Habitat type	Elevation (NAP)	Dominant vegetation	Large grazers	Orchestia presence	Cohesive layer depth core (cm)	Top layer from stable samples
**Dollard Bay**	do1	Silty established marsh	1.931	Festuca	cows	0	20	cohesive
	do2	Silty established marsh	1.898	Puccinelia	cows	0	20	cohesive
	do3	Silty established marsh	1.99	Phragmites	cows	1	20	cohesive
	do4	Silty pioneer marsh	1.396	Phragmites	ungrazed	1	20	cohesive
	do5	Silty tidal flat	0.986	\	ungrazed	0	20	\
**Holwerd**	ho1	Silty established marsh	1.546	Phragmites	ungrazed	1	20	cohesive
	ho2	Silty established marsh	1.638	Atriplex prostrata	ungrazed	1	20	cracks
	ho3	Silty established marsh	1.571	Atriplex prostrata	ungrazed	1	20	cohesive
	ho4	Silty pioneer marsh	1.149	Spartina	ungrazed	1	20	pioneer (mud)
	ho5	Silty pioneer marsh	1.141	Salicornia	ungrazed	0	20	pioneer (mud)
**Paulina**	pau1	Silty established marsh	2.413	Elytrigia atherica	ungrazed	0	20	cracks
	pau2	Silty established marsh	2.398	Elytrigia atherica	ungrazed	1	20	cracks
	pau3	Silty established marsh	2.338	A. portulacoides	ungrazed	0	20	cohesive
	pau4	Silty pioneer marsh	1.282	Spartina	ungrazed	0	20	cohesive
	pau5	Silty tidal flat	0.626	\	ungrazed	0	20	\
**Rilland**	ri1	Silty established marsh	3.406	Elytrigia atherica	ungrazed	0	20	detritus/lose
	ri2	Silty established marsh	3.004	Phragmites	ungrazed	0	20	pioneer (mud)
	ri3	Silty established marsh	3.042	Scirpus	ungrazed	1	20	pioneer (mud)
	ri4	Sandy pioneer marsh	2.738	Scirpus	ungrazed	1	0	\
	ri5	Sandy tidal flat	2.494	\	ungrazed	1	0	\
	ri6	Sandy tidal flat	2.304	\	ungrazed	1	0	\
**Ritt-hem**	rit1	Sandy pioneer marsh	2.019	Spartina	ungrazed	0	0	\
	rit2	Sandy pioneer marsh	1.933	Spartina	ungrazed	0	0	\
**Schiermonnikoog**	schi1	Sandy established marsh	2.222	Elytrigia atherica	ungrazed	1	0	cohesive
	schi10	Sandy established marsh	1.342	Aster	ungrazed	1	5	cohesive
	schi11	Sandy pioneer marsh	0.988	Salicornia	ungrazed	0	0	\
	schi12	Sandy tidal flat	0.857	\	ungrazed	0	0	\
	schi2	Sandy established marsh	1.801	Juncus maritimus	sheep	1	14.5	cohesive
	schi3	Sandy established marsh	1.834	Juncus maritimus	sheep	1	16	cohesive
	schi4	Sandy established marsh	1.866	Juncus gerardi	sheep	0	12.5	cohesive
	schi5	Sandy established marsh	1.521	Juncus maritimus	ungrazed	1	2	cohesive
	schi6	Sandy established marsh	1.147	Suaeda,Puccinellia	ungrazed	1	5	\
	schi7	Sandy pioneer marsh	1.129	Spartina	ungrazed	0	0	\
	schi8	Sandy established marsh	1.697	Elytrigia	ungrazed	1	10.5	cohesive
	schi9	Sandy established marsh	1.591	Juncus	ungrazed	1	6	\

**Uithuizen**	uit1	Silty established marsh	3.544	Festuca	sheep	0	20	cohesive
	uit2	Silty established marsh	3.587	Elytrigia atherica	sheep	0	20	cohesive
	uit3	Silty established marsh	3.322	Salicornia	sheep	0	20	cohesive
	uit4	Silty pioneer marsh	3.236	Salicornia	ungrazed	0	20	cohesive
	uit5	Silty tidal flat	3.143	\	ungrazed	0	20	\
**Waarde**	waa1	Silty established marsh	2.853	Limonium	ungrazed	1	20	cohesive
	waa2	Silty established marsh	2.891	Elytrigia atherica	ungrazed	1	20	detritus/lose
	waa3	Silty established marsh	2.848	Scirpus	ungrazed	1	20	cohesive
	waa4	Silty established marsh	2.983	Elytrigia atherica	ungrazed	1	20	cohesive
	waa5	Silty tidal flat	1.701	\	ungrazed	0	20	\
	waa6	Silty tidal flat	1.657	\	ungrazed	0	20	\
**Zuidgors**	zui1	Silty established marsh	2.374	Atriplex	ungrazed	1	20	cohesive
	zui2	Silty established marsh	2.602	Elytrigia atherica	ungrazed	1	20	cohesive
	zui3	Silty established marsh	2.441	Aster	ungrazed	0	20	cohesive
	zui4	Silty pioneer marsh	2	Spartina	ungrazed	0	20	pioneer (mud)
	zui5	Silty tidal flat	1.742	\	ungrazed	0	20	\
**Zwarte Haan**	zwa1	Silty established marsh	2.199	Festuca	cows	0	20	cohesive
	zwa2	Silty established marsh	1.925	Festuca	cows	0	20	cohesive
	zwa3	Silty established marsh	1.49	Suaeda	cows	1	20	cracks
	zwa4	Silty pioneer marsh	1.132	Spartina	ungrazed	0	20	pioneer (mud)
	zwa5	Silty pioneer marsh	1.145	\	ungrazed	0	20	\
**Griend**	gri1	Silty established marsh	1.381	Elytrigia	ungrazed	1	5.5	cohesive
	gri2	Silty established marsh	1.041	Atriplex	ungrazed	1	5	cohesive
	gri3	Silty established marsh	1.263	Aster	ungrazed	1	2.5	cohesive
	gri4	Sandy tidal flat	1.096	\	ungrazed	0	0	\

Table S2. Grain sizes from the top layer of the samples (0-5cm depth) determined with Malvern® Mastersizer 2000. SD50MUM_2 = Median grainsize D50 in µm, SD50PHIM_2 = Median grainsize D50 in PHI, SMODE_2 = Modus grainsize in µm, SCOARSE%_2 = Coarse sand fraction PHI 0-1, 500-1000 µm, SMEDIUM%_2 = Medium sand fraction PHI 1-2, 250-500 µm, SFINES%_2 = Fine sand fraction PHI 2-3, 125-250 µm, SVFINES%_2 = Very Fine sand fraction PHI 3–4, 62.5-125 µm, SSILT63_2 = Silt fraction < 63 µm

Code	SD50MUM_ 2 (µm)	SD50PHIM_ 2 (phi)	SMODE_ 2 (µm)	SCOARSE%_ 2 (%)	SMEDIUM%_ 2 (%)	SFINES%_ 2 (%)	SVFINES%_ 2 (%)	SSILT63_ 2 (%)
ri1	29.2	5.1	47.1	1.0	2.4	6.3	16.6	73.9
ri2	27.4	5.2	33.7	2.8	4.7	6.2	12.3	74.2
ri3	30.0	5.1	44.8	0.4	0.9	4.0	18.0	77.0
ri4	104.8	3.3	108.2	0.0	0.1	30.5	59.0	10.7
ri5	102.6	3.3	107.3	0.0	0.2	30.4	55.4	14.2
ri6	100.6	3.3	105.5	0.0	0.2	28.9	55.6	15.6
waa1	44.7	4.5	38.7	0.6	9.4	15.9	15.3	59.0
waa2	34.7	4.9	54.0	0.6	1.3	4.8	20.6	73.0
waa3	32.3	5.0	46.7	1.9	1.4	5.0	18.1	73.9
waa4	40.7	4.6	64.6	1.4	1.0	7.8	23.7	66.3
waa5	39.1	4.7	65.8	0.0	0.1	7.2	24.8	68.3
waa6	43.1	4.5	78.2	0.0	0.1	9.4	27.2	63.6
pau1	23.4	5.4	32.8	0.1	1.1	4.0	12.7	82.3
pau2	21.0	5.6	32.2	0.1	0.9	2.4	11.6	85.2
pau3	28.4	5.1	36.5	4.2	1.6	3.5	13.4	77.7
pau4	49.0	4.4	59.3	0.1	3.2	10.8	25.2	61.0
pau5	46.7	4.4	65.1	0.2	0.7	6.1	28.9	64.5
zui1	18.7	5.7	26.7	0.0	0.9	1.5	7.2	90.6
zui2	24.7	5.3	37.2	0.0	0.8	2.7	13.4	83.5
zui3	21.3	5.6	28.7	0.0	1.2	2.7	9.7	86.6
zui4	27.1	5.2	36.9	0.2	1.1	2.1	13.6	83.3
zui5	48.1	4.4	67.5	0.2	0.3	6.9	30.1	63.0
schi1	205.9	2.3	209.6	0.0	28.0	63.1	4.7	4.2
schi2	167.1	2.6	171.8	21.0	17.0	20.8	16.4	25.0
schi3	132.7	2.9	123.4	15.1	16.4	20.3	19.9	28.4
schi4	169.6	2.6	137.1	17.9	19.9	22.6	19.8	20.0
schi5	193.6	2.4	216.0	0.4	29.4	46.7	10.0	13.6
schi6	161.8	2.6	212.9	0.1	23.0	38.2	9.9	28.8
schi7	205.6	2.3	211.9	0.0	26.8	62.5	2.8	7.9
schi8	67.8	3.9	74.7	1.2	8.9	18.9	23.9	47.4
schi9	148.8	2.8	216.3	2.6	22.3	31.7	16.9	26.8
schi10	129.4	3.0	224.8	3.4	20.8	26.8	13.7	35.5
schi11	209.2	2.3	215.8	0.0	30.0	59.2	3.3	7.6
schi12	199.6	2.3	198.4	0.0	23.9	70.1	6.0	0.0
rit1	308.4	1.7	307.9	10.2	60.0	29.6	0.2	0.0
rit2	374.6	1.4	376.3	22.2	63.7	14.1	0.0	0.0

Do1	25.2	5.3	33.4	0.4	2.3	4.4	12.8	80.4
Do2	23.6	5.4	28.3	2.7	1.8	3.4	11.1	81.2
Do3	18.0	5.8	24.1	0.5	0.9	1.5	6.6	90.7
Do4	23.1	5.4	29.1	1.0	2.3	3.8	10.6	82.5
Do5	20.5	5.6	29.6	0.8	1.4	2.3	10.1	85.5
Uit1	25.8	5.3	32.5	0.4	1.6	3.6	12.6	82.0
Uit2	28.4	5.1	44.2	1.1	1.1	3.6	16.9	77.6
Uit3	38.5	4.7	55.8	0.8	1.6	5.0	22.4	70.5
Uit4	31.0	5.0	37.1	1.7	1.2	7.0	18.0	72.3
Uit5	23.5	5.4	28.6	0.1	0.4	1.6	10.0	88.2
Ho1	28.8	5.1	39.1	0.9	1.8	3.7	15.0	78.9
Ho2	23.6	5.4	30.9	1.5	1.5	3.0	11.3	82.9
Ho3	24.7	5.3	33.1	0.5	1.2	2.5	11.9	84.3
Ho4	23.0	5.4	30.3	0.0	0.2	0.7	9.4	89.8
Ho5	24.4	5.4	31.5	0.1	0.5	1.4	10.9	87.3
Zwa1	32.2	5.0	42.2	0.2	1.6	3.5	17.0	78.1
Zwa2	30.7	5.0	39.1	2.0	2.0	4.0	15.6	76.7
Zwa3	27.8	5.2	36.1	0.2	1.3	2.3	12.9	83.6
Zwa4	34.7	4.9	39.8	3.4	4.2	5.1	16.1	71.4
Zwa5	24.8	5.3	32.3	0.1	0.8	1.5	10.7	87.1
gri3	58.0	4.1	70.9	5.0	6.3	12.6	23.4	53.0
gri2	36.3	4.8	47.1	1.1	3.7	7.7	18.3	69.5
gri1	62.5	4.0	78.4	5.1	7.9	15.9	21.1	50.2
gri4	123.0	3.0	129.0	0.0	3.2	45.3	41.3	10.3

Fig. S1. Images from the method used to collect the rectangular soil samples for the erosion experiment.

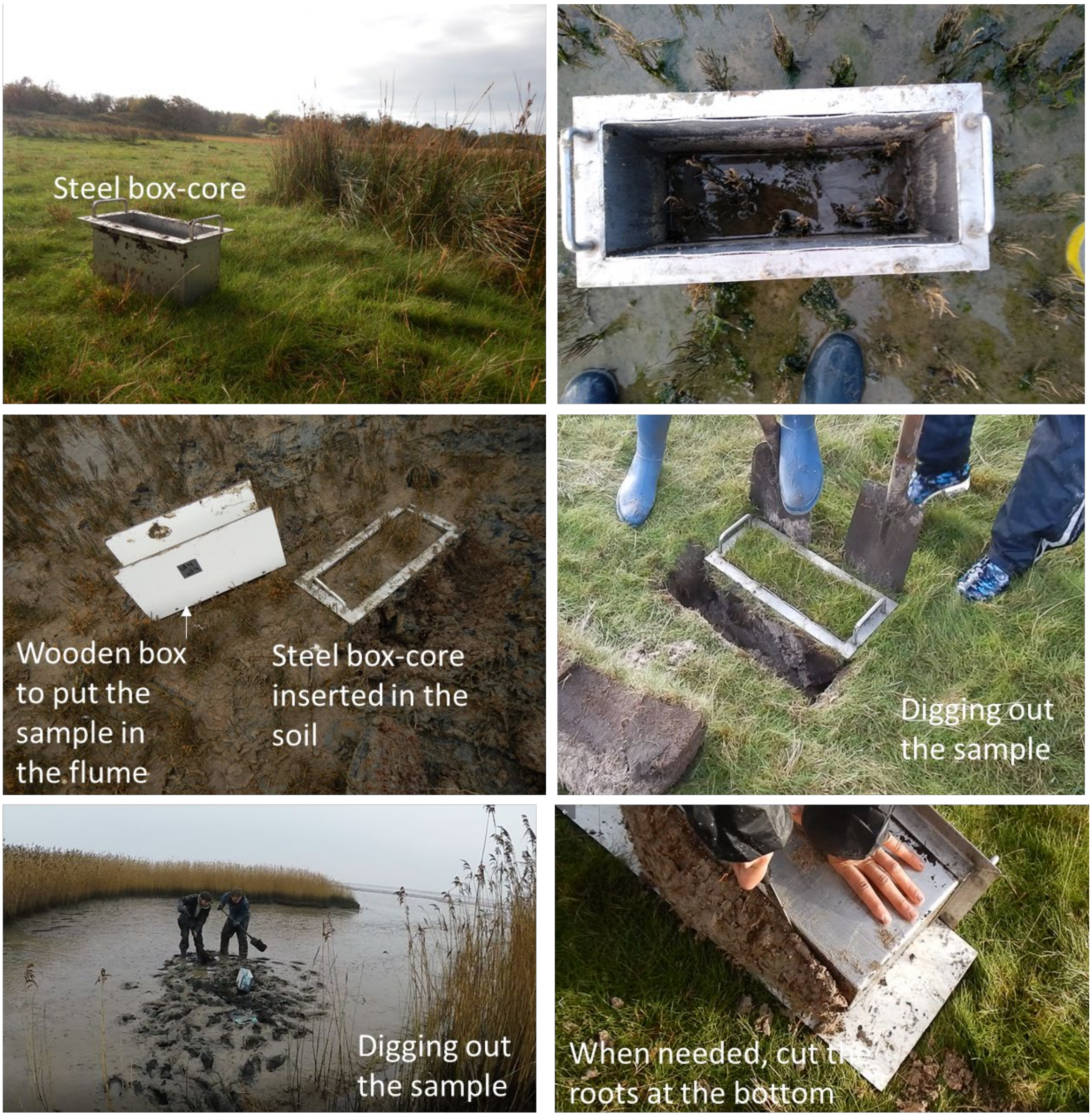

Fig. S2. a) Custom-made wooden boxes which fit in the flow flume with the soil samples inside, b) top view of a sample outside the flume. From each sample, 8 cm were cut off prior to the erosion test to analyse the vegetation belowground biomass and c) example of a sample inside the flume. An extra "wall" was installed in each side of the flume walls and on top of the sample edges to hold the samples and shield the small gap between the sample and the wooden box in order to prevent soil scouring in the margins of the sample. The extra walls were removed after each erosion test and installed again for every sample.

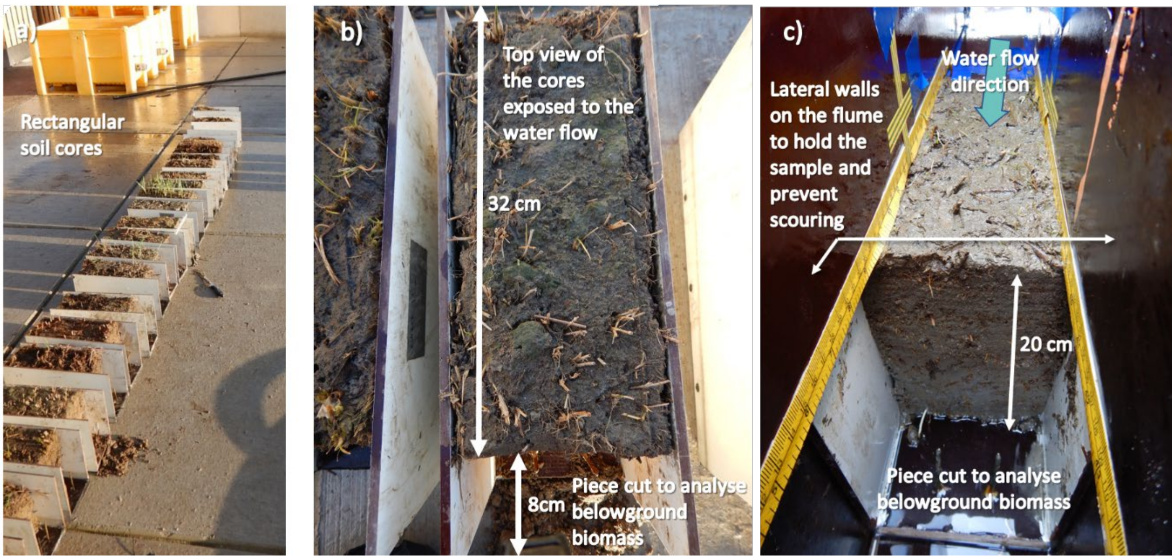

Fig. S3. Mean erosion depth over time from a subset of samples to test their stability during a prolonged period of time (16h) exposed to fast water flow. All these samples had an artificial crack made after 3h of flow exposure.

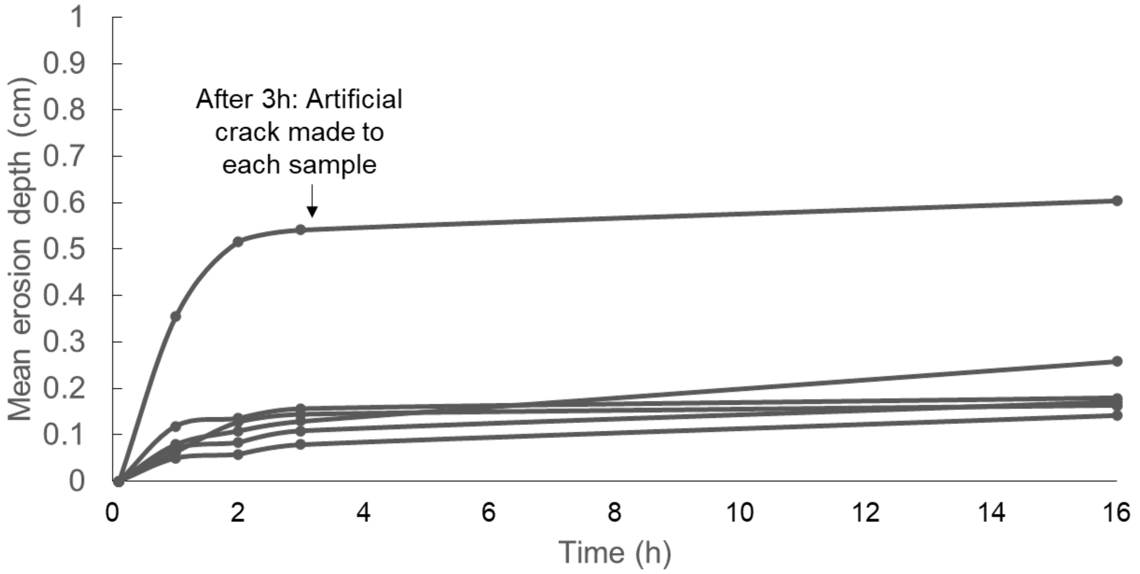

Fig. S4. Erosion data including all the samples, were it can be observed the binary response (completely eroded or not eroded). a) shows a boxplot of the mean top erosion, measured as erosion depth (cm), at the end of the experiment after 3 hours of flow exposure, separated by habitat types. The cores were 20 cm depth, therefore, erosion of 20 cm indicates complete erosion; b) shows the erosion depth (cm) in relation to the environmental variables measured.

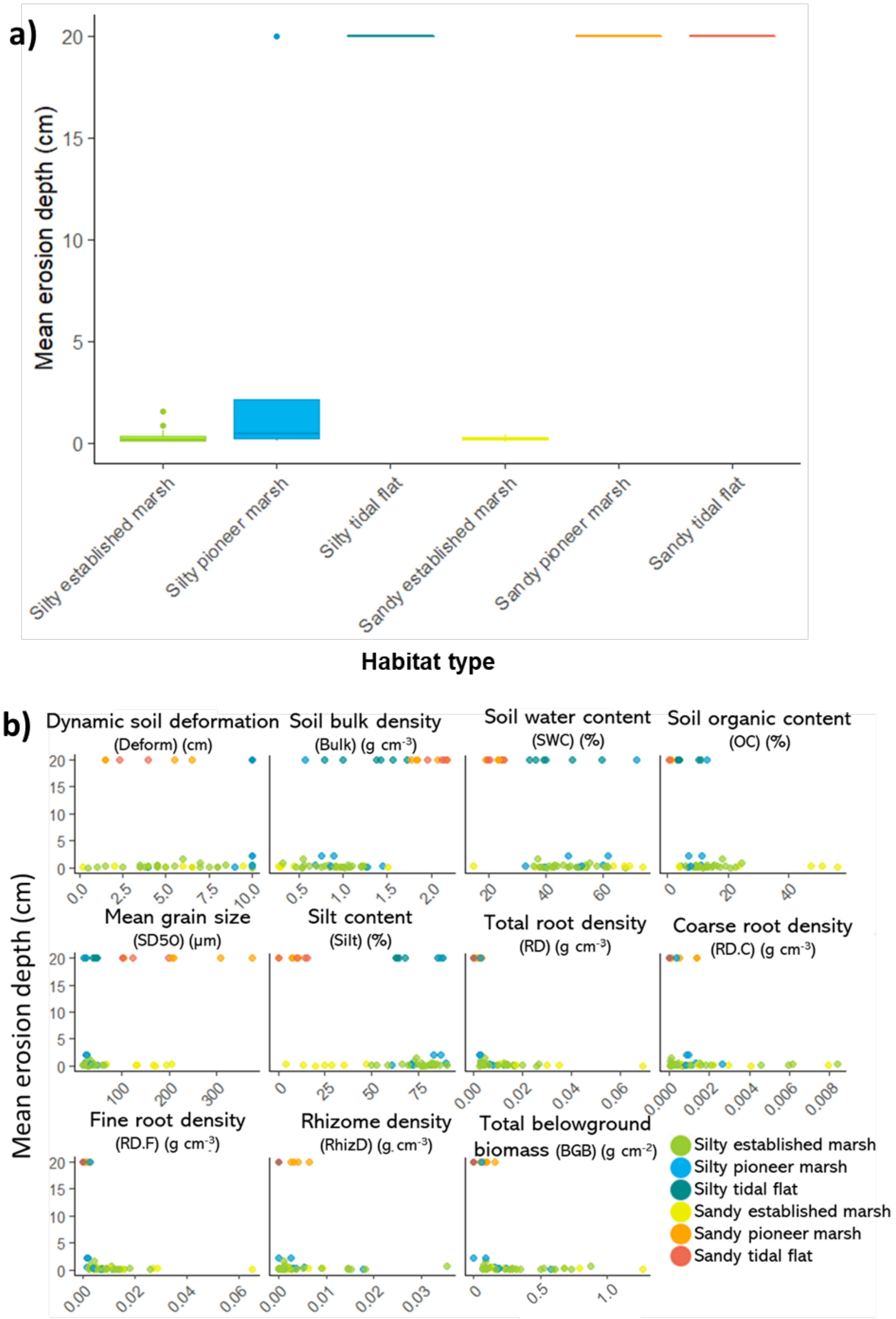

Fig. S5. Spearman correlation matrix showing the relationships among the environmental variables. Comp = dynamic soil deformation (cm), BulkD = Bulk density (g cm^{-3}), SWC = Soil water content (%), OC = Soil organic content (%), SD50 = mean grain size (µm), Silt = silt %, RD = total root density (g cm^{-3}), RhizD = Rhizome density (g cm^{-3}), RD.C = coarse root density (g cm^{-3}), RD.F = fine root density (g cm^{-3}), BGB = belowground biomass (roots + rhizomes) (g).

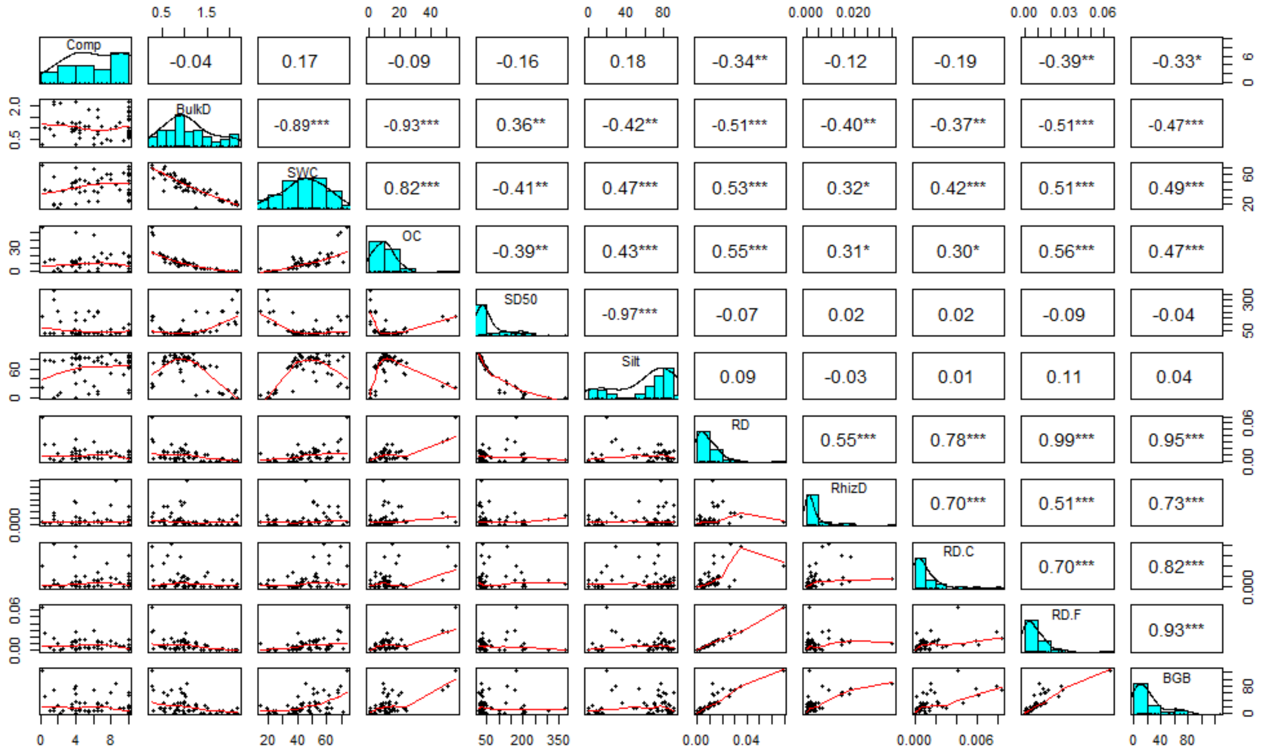

Fig. S6. Front view from a sandy tidal flat sample before and after 3 minutes of flow exposure, where it completely eroded; and from a sandy marsh with cohesive top layer, which did not erode after 3 hours of flow exposure.

Sandy tidal flat before erosion test

Sandy tidal flat, completely eroded after 3 min of flow exposure

Sandy marsh with cohesive top layer and sandy bottom, not eroded after 3h of flow exposure

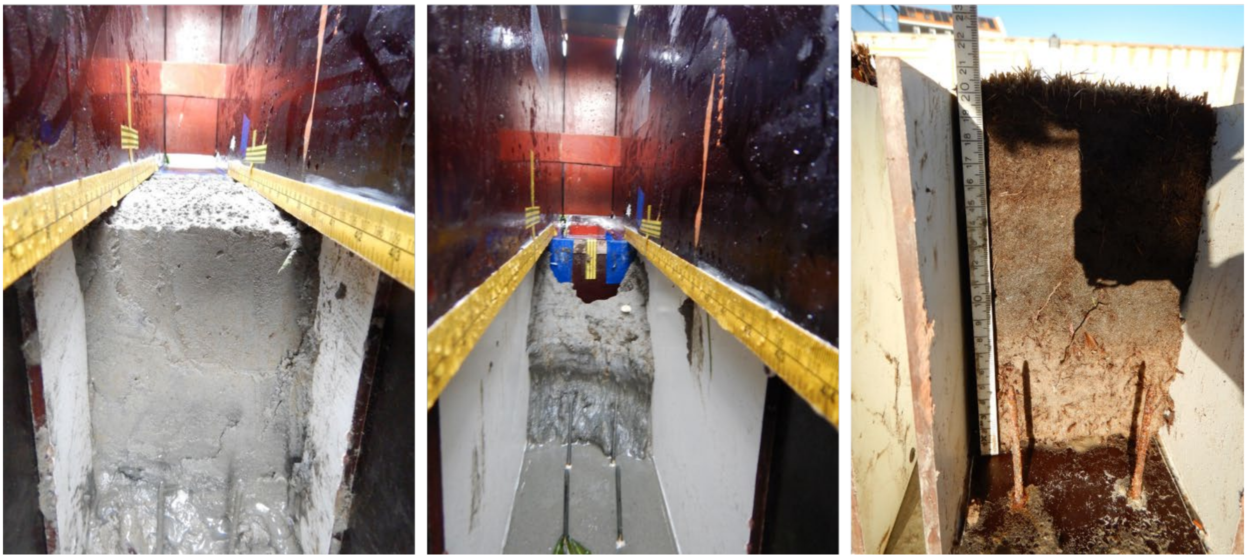

Fig. S7. Example of the erosion profiles measured with the erosion pins in a stable marsh sample and a bare tidal flat sample (not stable), for the two profiles done per sample.

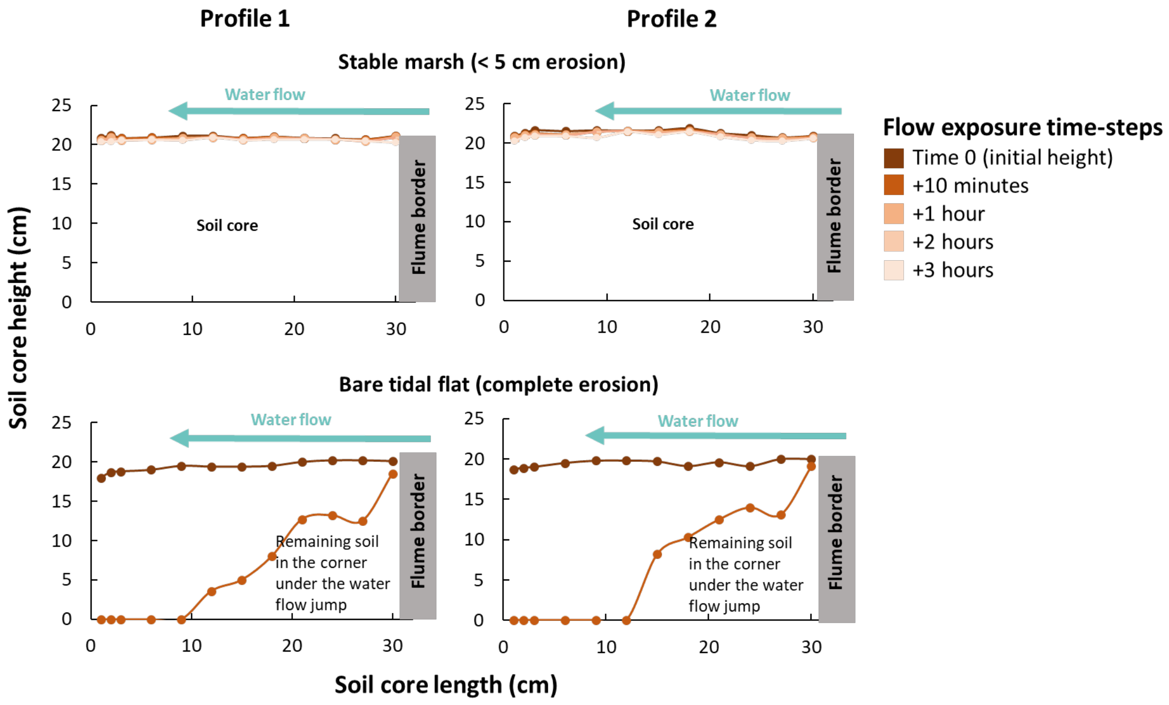

Fig. S8. Example of the erosion in form of blocks in samples with natural cracks due to a summer drought (top view of the sample).

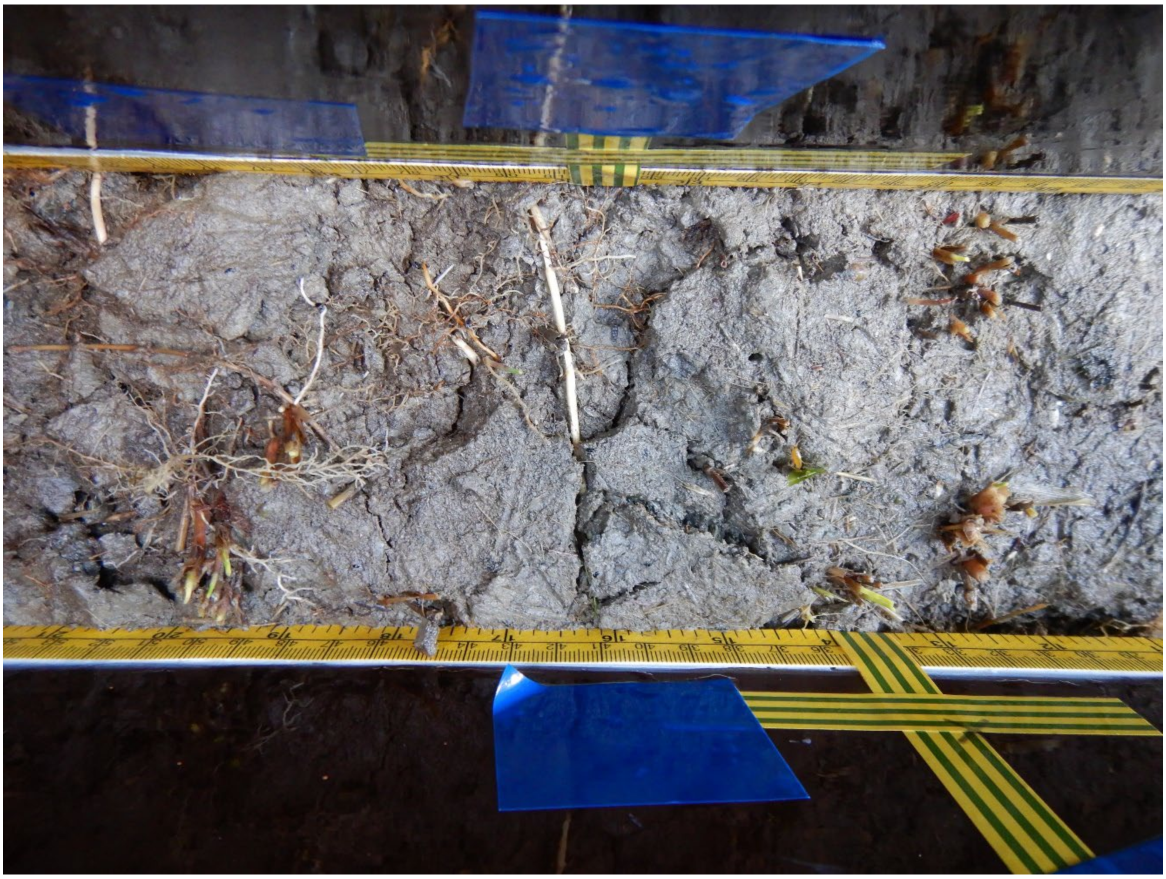

Fig. S9. a) Overview of the deep soil profiles up to 1.5 m depth found in the different habitat types from all the locations studied. Each bar corresponds to one sampling location. Silty and sand + peat top layers in the profiles are the ones considered cohesive top layers. It can be observed that it is common to find an alternation between silty and sandy layers. b) Deep profiles separated by habitat and location (Schi=Schiermonnikoog, ri=Rilland, rit = Ritthem, Gri=Griend, do=Dollard Bay, ho = Holwerd, Pau= Paulina, Uit= Uithuizen, Waa = Waarde, Zui= Zuidgors. Zwa = Zwarte Haan). More details from each location can be found in Table S1.

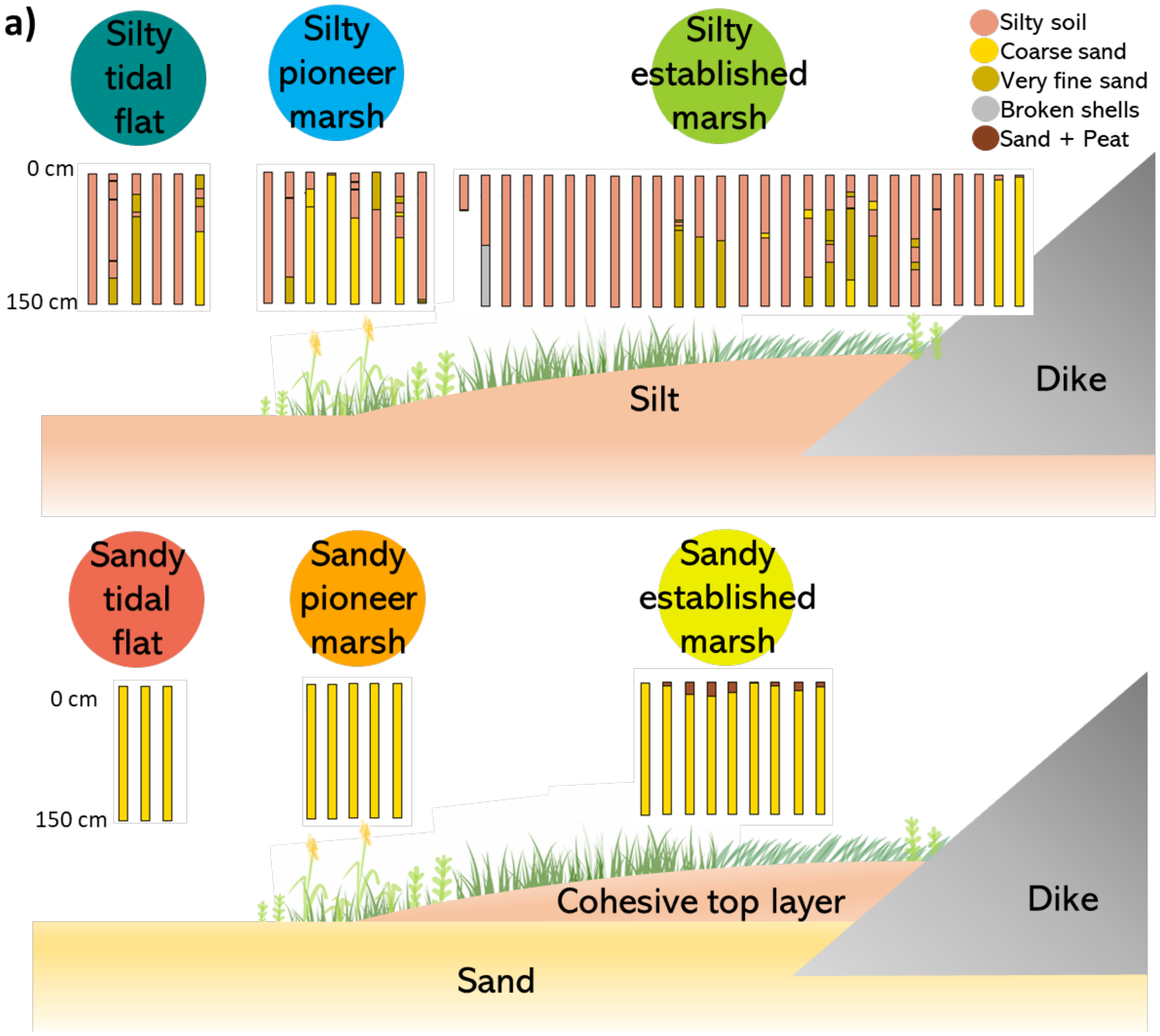

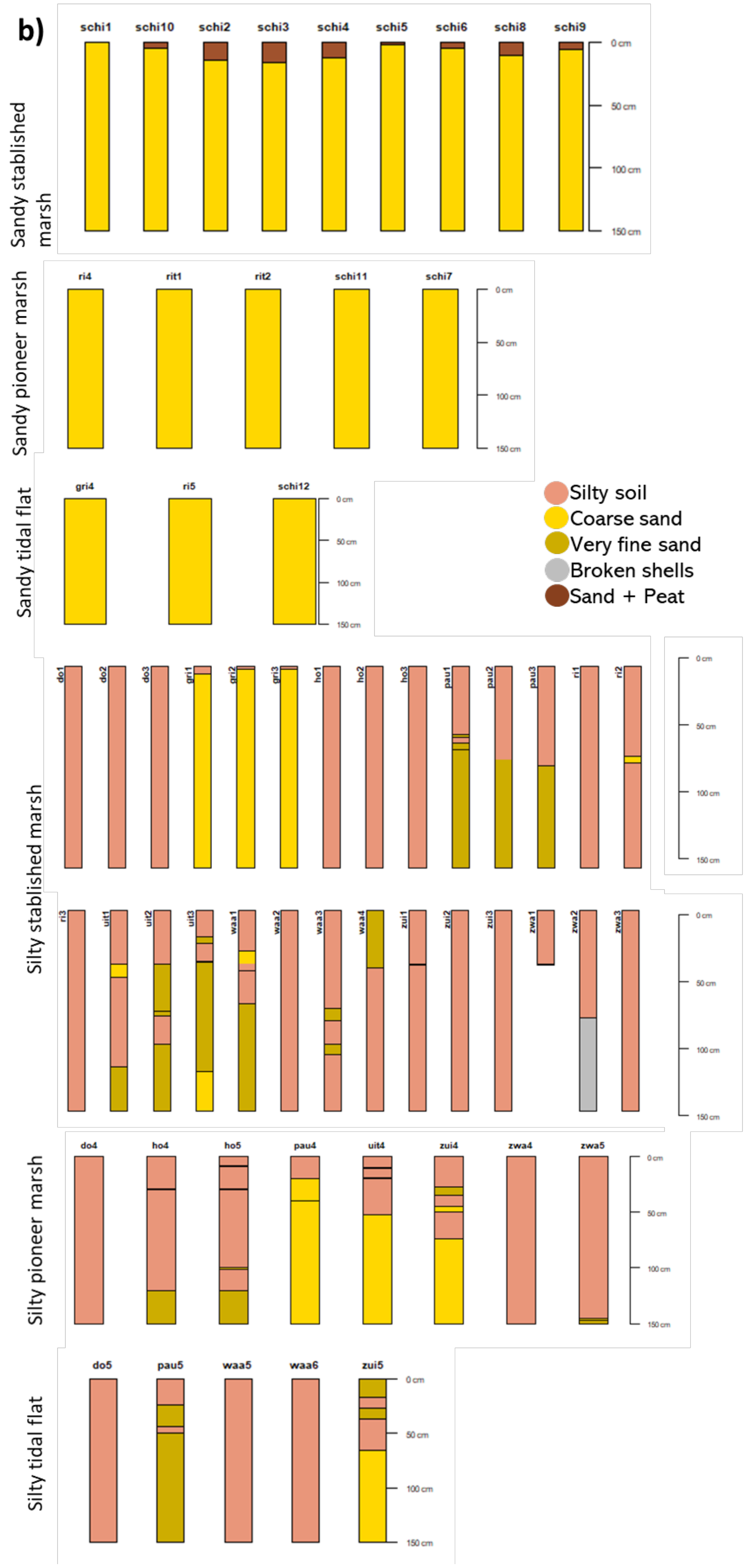
b)
Sandy stablished marsh
schi1
schi10
schi2
schi3
schi4
schi5
schi6
schi8
schi9
0 cm
50 cm
100 cm
150 cm
Sandy pioneer marsh
ri4
rit1
rit2
schi11
schi7
Sandy tidal flat
gri4
ri5
schi12
Silty soil
Coarse sand
Very fine sand
Broken shells
Sand + Peat
Silty stablished marsh
Silty pioneer marsh
do4
ho4
ho5
pau4
uit4
zui4
zwa4
zwa5
Silty tidal flat
do5
pau5
waa5
waa6
zui5
